# AI-Integrated Omics Analysis Reveals Cultivar-Specific Resistance Mechanisms to Powdery Mildew in *Cucurbita pepo*

**DOI:** 10.3390/ijms262311488

**Published:** 2025-11-27

**Authors:** Rita Dublino, Daniela D’Esposito, Anna Guadagno, Claudio Capuozzo, Paola Crinò, Gelsomina Formisano, Maria Raffaella Ercolano

**Affiliations:** 1 Department of Agricultural Sciences, University of Naples Federico II, Via Università 100, Portici, 80055 Naples, Italy; rita.dublino@unina.it (R.D.); desposito.daniela@unina.it (D.D.); anna.guadagno@unina.it (A.G.); capuozzo.claudio@unina.it (C.C.); 2 ENEA Casaccia Research Centre, Via Anguillarese 301, 00123 Rome, Italy; paola.crino@enea.it; 3 La Semiorto Sementi S.r.l. Sarno, Via Vecchia Lavorate 81-85, Sarno, 84087 Salerno, Italy; ricerca@semiorto.com

**Keywords:** *Cucurbitaceae*, *Podosphaera xanthii*, omics-analysis, transcriptome analysis, functional genomics, resistance strategies, clustering algorithms, unsupervised machine learning

## Abstract

Powdery mildew represents one of the most significant challenges for cucurbit crops. In recent decades, progress has been made in identifying resistance sources that improve yield and quality while reducing fungicide use. This study explored the molecular mechanisms underlying cucurbit responses to powdery mildew through comparative RNA-seq of two contrasting *Cucurbita pepo* cultivars: the partially resistant 968Rb and the susceptible True French. Differential expression analysis between inoculated and non-inoculated conditions identified 398 DEGs in 968Rb and 1129 in True French. In 968Rb, a stronger defense response emerged with cell wall reinforcement and upregulation of *fructose-1,6-biphosphate aldolase* genes, while True French showed activation of chitinase genes. Machine learning models, including Random Forest and K-means, identified expression features and gene modules linked to resistance. By combining conventional and Artificial Intelligence-based analyses, we identified a putative adaptive genetic variation, supported by a higher single nucleotide polymorphism density within expression clusters enriched for upregulated genes in the partial resistant cultivar 968Rb. The integration of Artificial Intelligence tools in our pipeline facilitated the understanding of the genetic basis of *Cucurbita pepo* resistance to *Podosphaera xanthii*, highlighting the transcriptional modules and variant patterns associated with resistance traits, and providing a scalable framework for future applications in crop improvement.

## 1. Introduction

*Cucurbitaceae* species are important vegetable crops worldwide with an estimated annual production of over 20 million tons [1]. This family includes nearly 1000 plant species known universally as cucurbits [2]. Among them, *Cucurbita pepo* L. is a valuable vegetable crop widely cultivated in many countries for its nutritional, economic, and cultural value [3].

Powdery mildew (PM) is a very important disease for all *Cucurbitaceae* crops, reducing yield potential and fruit quality [4]. In particular, the fungal pathogen *Podosphaera xanthii* is the main PM causal agent in the Mediterranean area. The genetic sources of resistance to PM in cucurbits are fundamental for enhancing crop yields, improving quality, and reducing dependency on fungicides.

Different physiological *P. xanthii* races have been described worldwide on cucurbits, identified through differential host assays, and their distribution can vary across years, regions, and hosts [5]. Studies conducted in southern Europe, including Italy, revealed the coexistence of multiple races in *C. pepo*, such as races 1, 3, 4, 5, and 2FR, with clear differences in prevalence among cucurbit species [6]. Notably, in *C. pepo*, isolates frequently belonged to races 1, 4, and 5, reflecting a high diversity of virulence profiles even within local pathogen populations. This race structure has important implications for disease management and for the development of resistant cultivars, given the capacity of *P. xanthii* populations to rapidly shift under environmental and agronomic pressure.

Although significant advances in biotic stress resistance have been made in zucchini over the past twenty years, knowledge about mildew resistance in *C. pepo* remains limited and is progressing slowly compared to other cucurbit species. The availability of omics methodologies has allowed researchers to explore the molecular basis of resistance and susceptibility in different species. Genome-wide association studies (GWAS) have identified several key genomic regions linked to powdery mildew resistance in *Cucumis melo* and *C. moschata* [7,8]. Moreover, transcriptomic studies have revealed distinct transcriptomic profiles in different cultivars in cucumber or melon inbred lines. These works elucidated defense response mechanisms through transcriptome sequencing, highlighting the importance of specific DEGs in conferring resistance to powdery mildew [9,10,11]. Despite advances in genomic analysis, the intricate patterns of response of *C. pepo* to PM pose interpretative challenges. In this context, the integration of artificial intelligence (AI) with conventional omics analyses is increasingly recognized as a revolutionary strategy in modern biology. Recent works indicate the success of machine learning (ML) and deep learning (DL) in genomics and spatial transcriptomics, with evidence of improvements in variant calling and pathway analyses [12,13]. ML methods such as Random Forest (RF), K-means, and Self-Organizing Maps (SOM) were able to identify significant features in high-dimensional gene expression data while providing insight into complex, non-linear relationships among genes [14,15]. DL strategies helped to capture genotype–phenotype relationships that are not easily identifiable through traditional analytical pipelines. By combining AI-based analysis of gene expression with SNP/InDel discovery, recent studies have elucidated how genetic variation contributes to complex transcriptional responses [16]. Integrative approaches have been effectively applied in cancer precision medicine [17,18] and tropical disease diagnosis [19], highlighting the ability of these methods to reduce bias and improve interpretability. These findings have supported the extension of integrative approaches to plant systems, particularly in the study of disease resistance traits.

The aim of this study is to reveal the genetic differences between two near-isogenic cultivars of *C. pepo*, the partially resistant 968Rb and the susceptible True French (TF). These lines were developed through six back-cross generations, resulting in high genetic similarity (>0.80), with divergence primarily in regions associated with powdery mildew resistance [20]. A specific analytical pipeline combining traditional bioinformatics and AI-driven techniques has been set up. Transcriptomic profiles of the inoculated and uninoculated cultivars were obtained using conventional analytical software. AI tools supported the interpretation of the genotype-specific response and the reconstruction of hidden gene regulatory networks and the analysis of transcript variants underlying defense traits. The applied integrative methodologies underline their critical role in advancing both basic research and translational applications in crop improvement.

## 2. Results

### 2.1. Podosphaera xanthii Inoculation and Disease Progression

The severity of *P. xanthii* disease was evaluated on inoculated *C. pepo* leaves using a 0–3 scale where 0 indicates no infection and 3 corresponds to full infection. Three days post-inoculation, the susceptible genotype (TF) exhibited both higher disease severity score and a greater number of leaves infected with *P. xanthii* compared to the partially resistant genotype (968Rb); a visual comparison is provided in Supplementary Figures S1 and S2. By the final disease evaluation, all TF leaves (38/38) were fully infected, displaying dense powdery mildew coverage, whereas 968Rb leaves (45/45) remained only partially colonized. The mean percentage of leaf area covered differed markedly between the two genotypes, reaching 48.2% in TF compared to 23.5% in 968Rb, confirming the reduced susceptibility of 968Rb. According to Duncan’s multiple range test, the difference between the two means was statistically significant, supporting the presence of a distinct phenotypic reaction to *P. xanthii.*

### 2.2. Transcriptomic Analysis and Key Classification Features

**Figure 1:**
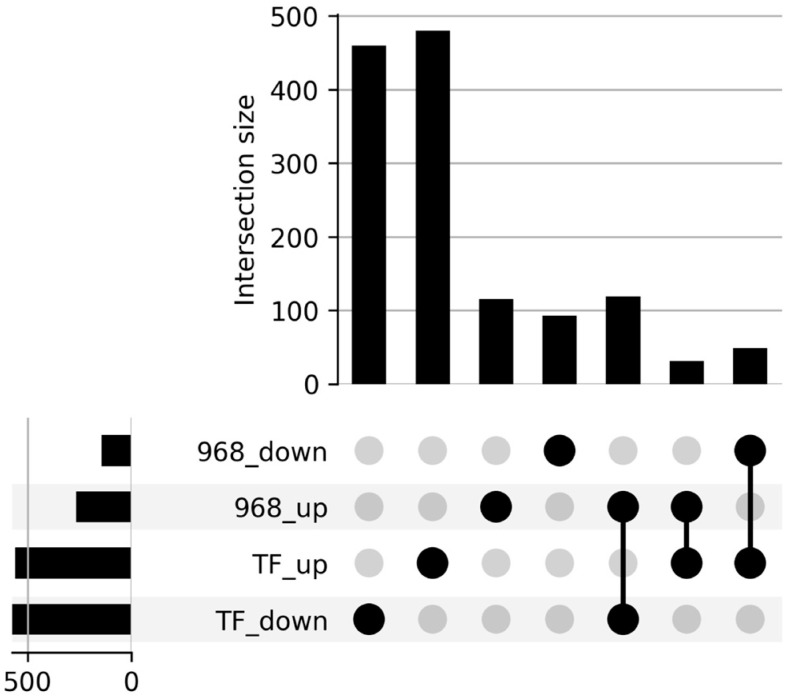
UpSet plot displaying DEGs intersections in *C. pepo* cultivars. The plot highlights unique and shared DEGs between the resistant cultivar 968Rb and the susceptible cultivar TF following *P. xanthii* inoculation. Horizontal bars on the left indicate the total number of DEGs within each set. Black filled dots identify the sets involved in a given intersection and are connected by vertical lines, whereas light gray dots indicate non-inclusion. Vertical bars at the top represent the number of genes within each intersection. Up/down labels refer to genes that were up- or down-regulated in each cultivar.

The sequencing of 12 libraries obtained from two *C. pepo* cultivars (968Rb and TF) inoculated and not inoculated with *P. xanthii*, generated ~19 million paired end reads per sample (Supplementary Table S1). Approximately 24,000 transcripts from two cultivars were mapped to the reference genome of *C. pepo* (v4.1). DEGs analysis between inoculated and not-inoculated samples was performed to better understand the response of *C. pepo* cultivars to *P. xanthii* infection. A total of 398 DEGs were identified in 968Rb and 1129 in TF, respectively; 260 genes were upregulated in the 968Rb genotype, while 554 were upregulated in the TF genotype. On the other hand, 138 genes were downregulated in 968Rb and 575 were downregulated in TF (Supplementary Tables S2 and S3). An UpSet plot (Figure 1) was generated to visualize the intersection between the differentially expressed gene sets. This analysis revealed that 114 genes were exclusively upregulated in 968Rb, and 91 exclusively downregulated. For the TF cultivar, 478 genes were uniquely upregulated and 458 uniquely downregulated. Crucially, 117 genes were upregulated in the partially resistant 968Rb, but simultaneously downregulated in the susceptible TF, while 47 genes showed an opposite expression pattern. Furthermore, 29 genes were upregulated in both cultivars. No genes were found to be downregulated in both genotypes.

### 2.3. Classification of Gene Expression Data Using Random Forest Models Random Forest Classification of Variables

**Figure 2:**
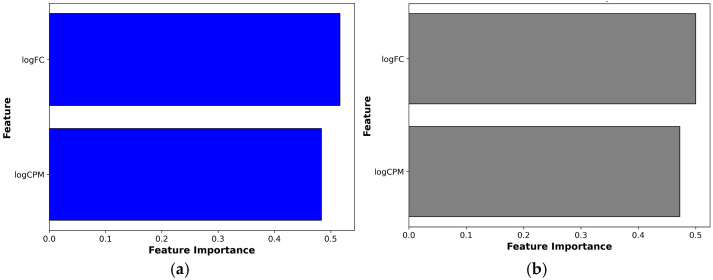
Feature Importance analysis of DEGs classification. Panel (**a**) shows the Feature Importance results from the RF model, where the bars represent the contribution of each feature (logFC and logCPM) to the classification task. Panel (**b**) illustrates the results of the Permutation Feature Importance test, where the length of each bar indicates the mean decrease in model performance when that specific feature’s values are randomly shuffled, highlighting its importance for accurate classification.

To explore the contribution of key expression metrics to the classification of DEGs between the two *C. pepo* cultivars, an exploratory RF model was implemented. Initially, several variables from the differential expression analysis were considered, including logarithm of fold change (logFC), logarithm of counts per million (logCPM), *p*-value, F-value (F), and false discovery rate (FDR). After evaluating their relative importance, logFC and logCPM emerged as the most informative descriptors for distinguishing DEGs across experimental groups and were therefore selected for the final model. The RF model yielded nearly equal importance scores (51.6% for logFC and 48.4% for logCPM; Figure 2a), indicating that both the extent of transcriptional regulation and the basal expression level contribute to the observed transcriptomic divergence between cultivars. Their complementary interpretive roles were further supported by a permutation-based significance analysis, which showed that changing either variable significantly reduced the model performance (Figure 2b). The Permutation Feature Importance results confirmed that both dimensions of expression are essential to understand cultivar-specific transcriptional responses to *P. xanthii* infection.

### 2.4. AI-Based Clustering and Interpretation of DEG Patterns

**Figure 3:**
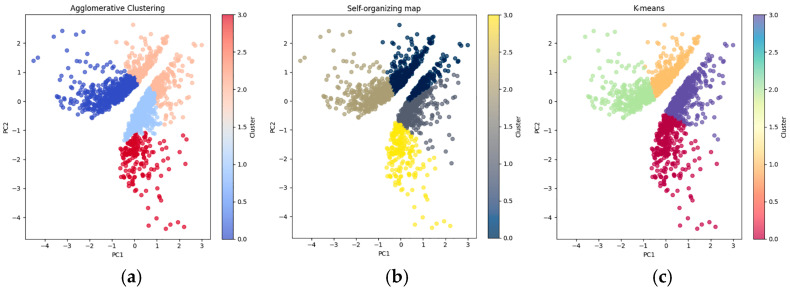
PCA visualizations of clustering results. The plots show the clustering results obtained by three different clustering algorithms: Agglomerative Clustering (**a**), Self-Organizing Map (**b**), and K-means (**c**). Each plot represents the distribution of the data along the first two principal components (PC1 and PC2) after applying PCA for dimensionality reduction. The colors indicate the different clusters identified by each algorithm.

Unsupervised machine learning methods were used to cluster DEGs based on their expression profiles, to reveal underlying transcriptional patterns and further stratify the response to infection. The clustering results obtained from Agglomerative Clustering, SOM, and K-means were visualized through PCA, which enabled a two-dimensional representation of the data as reported in Figure 3. The PCA plots provided an overview of clustering performance of each method. Agglomerative Clustering and SOM displayed some overlap between clusters, light blue and red clusters for the agglomerative method (Figure 3a) and gray and blue for the SOM (Figure 3b), suggesting that these methods can group similar data points, but not resolve all local proximities. Such overlap may highlight more complex interactions among expression profiles. On the other hand, K-means’ centroid-based separation strategy, reducing the within-cluster variation, showed a clear distinction between the four clusters (Figure 3c).

To quantitatively evaluate the performance of each clustering method, three commonly used validation indices were calculated: the Adjusted Rand Index (ARI), the Calinski–Harabasz Index (CHI), and the Davies–Bouldin Index (DBI) (Table 1). The ARI, which measures the agreement between the predicted clusters and a reference classification, adjusting for random chance, showed notable differences in performance between the methods. It assessed how well each method separated expression patterns across experimental groups. The SOM algorithm achieved the highest ARI (0.5491), followed by Agglomerative Clustering (0.5037), and K-means (0.4142), indicating that SOM captured the experimental group structure more effectively. The CHI evaluates the ratio of between-cluster dispersion to within-cluster compactness; higher values indicate more distinct and compact clusters. K-means obtained the highest CHI value (1171.0), suggesting optimal separation and compactness. The DBI estimates the average similarity between each cluster and its most similar one, where lower values indicate better clustering. Again, K-means performed best (0.8430), followed by SOM (0.9082) and Agglomerative Clustering (0.9368). Overall, K-means outperformed the other clustering methods in terms of cluster separation and compactness, as indicated by the highest CHI and the lowest DBI. While SOM achieved a higher ARI, suggesting better preservation of genotype-specific expression structures, K-means still produced more distinct and compact clusters overall. Based on these observations, K-means provided the cleanest and most interpretable results and was selected for further analyses. The ideal number of K for separating data with K-means, calculated using the Silhouette Score (0.56), was 4 (Supplementary Table S4). This result was visually confirmed by the PCA (Figure 3 right) which identifies the following: Cluster 1 (Purple), including strongly upregulated genes, with high values of logFC and logCPM; Cluster 2 (Green), including genes that are strongly downregulated, with low logCPM and negative logFC values; Cluster 3 (Blu), which includes genes showing moderate downregulation in expression, with negative logFC values and relatively higher logCPM; Cluster 4 (Yellow), containing genes that are moderately upregulated (high logFC and logCPM). In conclusion, K-means not only performs better than other clustering algorithms, but it also produces an extremely comprehensible output that shows separation of the DEGs into four distinct clusters and makes it easy to distinguish between upregulated and downregulated genes to arrange the further analysis.

### 2.5. Cluster Distribution and Expression Dynamics in Contrasting Genotypes

**Figure 4:**
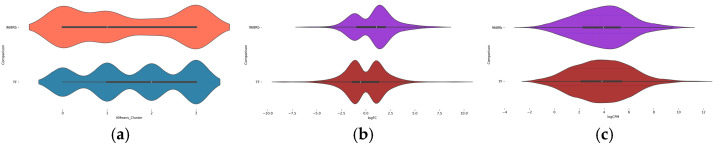
Violin plot comparison of DEG distributions and expression features between cultivars. Violin plots show comparison of DEGs across the different clusters in the two cultivars (**a**) and the selected key features: logFC (**b**) and logCPM (**c**).

To gain insight into the biological meaning of clustering, the expression profiles of DEGs were analyzed in relation to their distribution across the four K-means clusters and compared between the two cultivars by using the selected features, logFC and logCPM, as key transcriptomic indicators from RF (Figure 4). Violin plots revealed clear differences in the distribution of DEGs between 968Rb and True French. Figure 4a showed that 968Rb DEGs are distributed across all clusters, with a higher concentration in clusters 1 and 4, while TF is more concentrated in clusters 2 and 3. A distinct difference in gene expression is shown comparing the logFC values of 968Rb and TF (Figure 4b). 968Rb shows a larger distribution of logFC values, including both positive and negative values. In contrast, TF exhibits a more restricted distribution of logFC values, with predominantly negative values, reflecting a limited response. Also, 968Rb exhibits higher logCPM values than TF (Figure 4c), indicating a more pronounced reaction to infection. Despite having fewer DEGs, 968Rb shows a more efficient and targeted defense response, characterized by broader gene expression variability and greater overall activity. By contrast, TF showed a more limited and less dynamic gene response.

### 2.6. Global Functional Interpretation of Transcriptomic Clusters via GPT-4

Each transcriptomic cluster was functionally interpreted using a multi-step approach that combined GO enrichment analysis with GPT-4 based annotation. For each cluster and for each cultivar, GPT-4 generated a descriptive biological title and a concise functional summary, based on the most enriched GO terms (FDR < 0.05). This strategy allowed the identification of distinct biological programs associated with each expression pattern, such as early immune activation, cell wall reinforcement, or oxidative stress adaptation. The expression direction (up- or downregulation), cultivar specificity, and AI-generated interpretations are summarized in Table 2, while the full list of GO terms and functional descriptions is available in Supplementary Table S5.

### 2.7. MapMan Analysis to Refine DEGs Pathway Associations

**Figure 5:**
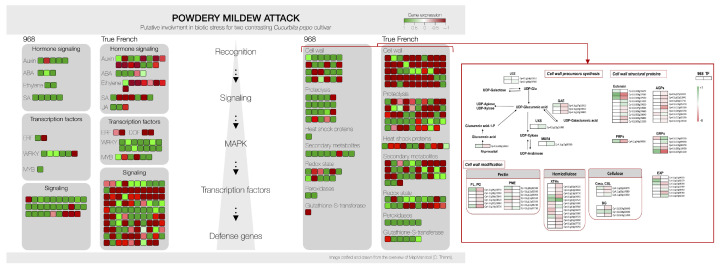
DEGs and cell wall metabolism overview in the two *C. pepo* cultivars following *P. xanthii* infection. Schematic overview of differentially expressed genes (DEGs) in partially resistant (968Rb) and susceptible (TF) *C. pepo* genotypes following *P. xanthii* inoculation. Genes are categorized based on functional annotation using MapMan, with expression changes indicated by color (green: upregulated; red: downregulated). The figure includes a focused view of DEGs involved in cell wall metabolism, highlighting genes related to precursor synthesis (pectin, hemicellulose, cellulose), cell wall modification, and structural proteins. Key gene abbreviations are provided to assist interpretation (e.g., *UGE, UXS, MUR4, GAE, PL, XTHs, CSL*). This integrated representation illustrates both the overall transcriptional response to biotic stress and specific alterations in cell wall-related pathways relevant to disease resistance.

A comprehensive overview of differentially expressed genes in both genotypes across cellular compartments and biological processes was provided by MapMan analysis. Pathways associated with signaling, cell wall, proteolysis, transcription factor, hormone signaling, and secondary metabolites resulted in being highly represented. As shown in Figure 5, hormone signaling pathways were activated in both genotypes but exhibited distinct patterns. In 968Rb, almost all hormones, except jasmonic acid (JA), were highly activated. Salicylic acid (SA), a key hormone of defense response against plant biotrophic pathogens [21], was particularly upregulated in 968Rb, indicating that this hormone plays a pivotal role in the resistance mechanism of this genotype. Conversely, in True French, there was a predominance of JA and abscisic acid (ABA) signaling related to defense and stress responsive gene expression. In the partially resistant cultivar 968Rb, defense-related transcription factors (TFs), including the WRKYs, MYBs, and ERFs, showed increased expression. By contrast, in True French, a downregulation of ERFs and DOFs and a pronounced upregulation of WRKY contributed to the genotype’s susceptibility to infection. The two genotypes also showed differences in the expression of genes involved in the cell wall metabolism and composition, including the synthesis of cell wall precursors, the structural proteins, and the genes involved in the modification of cell wall components (Figure 5). An upregulation of genes involved in the formation of UDP-sugars was observed in TF, while among the genes contributing to glucuronic biosynthesis (Cp4.1LG03g14330) displayed contrasting expression patterns between 968Rb and TF, supporting its potential role in cultivar-specific cell wall remodeling. In addition, three genes involved in the interconversion between UPD-glucuronic acid and UDP-galacturonic acid were downregulated in TF, whilst one UDP-D-glucuronate 4-epimerase (Cp4.1Lg17g02910) resulted in being upregulated in 968Rb. A widespread downregulation of genes involved in the modification or synthesis of key cell wall components, including pectin, hemicellulose, and cellulose, was observed in TF. By contrast, an opposite trend in the expression of genes encoding xyloglucan endotransglucosylases/hydrolases was observed. Cellulose metabolism was strongly activated in 968Rb while TF also included downregulated genes. A strong positive regulation of genes encoding structural proteins such as extensins, proline-rich proteins, and glycine-rich proteins was also observed in 968Rb.

### 2.8. Functional Categorization of DEGs Through GO and KEGG Analysis

**Figure 6:**
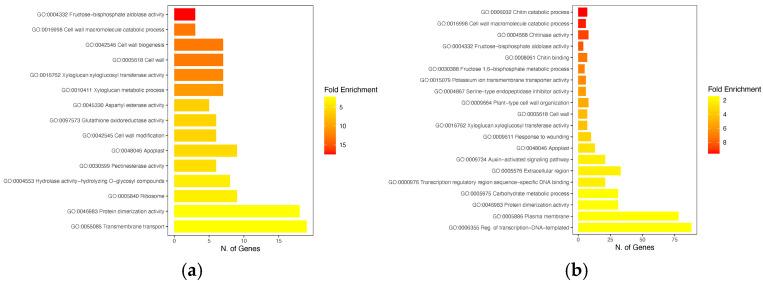
Functional categorization of DEGs in 968Rb and True French based on GO analysis. The functional enrichment of transcripts based on gene ontology (GO) categorization is shown. The graph displayed the most abundant GO terms identified using a false discovery rate (FDR) method for *p*-value correction in 968Rb cultivar (**a**) and TF cultivar (**b**). Only GO terms with a *p*-value ≤ 0.05 after correction are considered significantly enriched and are shown. The x-axis indicates the number of genes within each main category represented on the y-axis. The bars are color-coded according to the size of fold enrichment: red indicates high value of fold enrichment, orange indicates intermediate values, while yellow indicates low fold enrichment.

Gene ontology (GO) analysis allowed the assignment of specific GO terms to DEGs in biological process, molecular function, and cellular component categories. The enriched GO terms for each genotype, belonging to the three main categories (FDR < 0.05), are shown in Figure 6a,b. The partially resistant genotype showed several enriched GO terms related to “Cell wall” and “Cell wall biogenesis-modification”. The most enriched term in 968Rb (GO:0004332) is associated with the genes Cp4.1LG02g00380, Cp4.1LG03g01130, and Cp4.1LG05g12880 (Figure 6a). It is worth noting that this GO term is also enriched in the susceptible cultivar TF (Figure 6b), due to the same genes (except for Cp4.1LG05g12880, which is replaced by Cp4.1LG13g06960). Among these genes, the Cp4.1LG02g00380 (*FBA gene—Fructose-1,6-biphosphate aldolase*), which plays a critical role in stress response, is upregulated in 968Rb and downregulated in TF.

**Figure 7:**
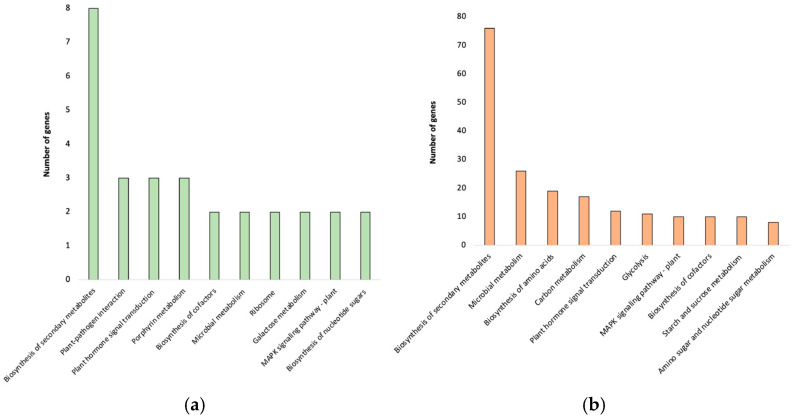
KEGG pathway enrichment analysis of transcripts from *C. pepo* cultivars. This figure shows the results of separate KEGG pathway enrichment analyses performed on transcripts identified from two cultivars: 968Rb (**a**) and True French (**b**). The analyses identified the ten most significantly enriched KEGG pathways within the KEGG database. The x-axis denotes the specific KEGG pathway identified. The y-axis represents the number of transcripts assigned to each enriched pathway.

In the susceptible cultivar TF, the enriched term related to chitin catabolism (GO:0006032) is associated with six genes. Among them, the Cp4.1LG10g10530 gene resulted in being upregulated in TF and downregulated in 968Rb. The term (GO:0016762) related to cell wall biology (Xyloglucan: xyloglucosyl transferase activity) is enriched in both the susceptible (TF) and partial resistant (968Rb) cultivars. The Cp4.1LG09g05000, Cp4.1LG16g04470, and Cp4.1LG16g05080 genes resulted in being specifically associated with 968Rb whilst Cp4.1LG01g09090, Cp4.1LG15g07730, and Cp4.1LG18g06250 with TF. A total of 252 KEGG pathways (FDR-corrected *p*-value threshold of ≤0.05) resulted in being enriched by the identified DEGs, of which 218 were associated with the TF cultivar and 45 with the 968Rb cultivar. A total of six KEGG pathways were enriched in both cultivars (Figure 7a,b) including “biosynthesis of secondary metabolites” (ko01110), “plant hormone signal transduction” (ko04075), “plant-pathogen interaction” (ko04626), “biosynthesis of cofactors” (ko01240), “microbial metabolism in diverse environments” (ko01120), and “MAPK signaling pathway” (ko04016).

However, the analysis also highlighted pathways unique to each cultivar, suggesting distinct metabolic strategies in response to the pathogen. The partial resistant cultivar 968Rb showed specific enrichment in pathways such as porphyrin metabolism (ko00860), ribosome (ko03010), galactose metabolism (ko00052), and biosynthesis of nucleotide sugars (ko01250). The enrichment in porphyrin metabolism and ribosome pathways suggests an increased synthesis of new proteins and a potential modulation of photosynthesis. The susceptible cultivar TF displayed unique enrichment in pathways related to the biosynthesis of amino acids (ko01230), glycolysis/gluconeogenesis (ko00010), carbon metabolism (ko01200), amino sugar and nucleotide sugar metabolism (ko00520), and starch and sucrose metabolism (ko00500). Pathways involved in amino sugar and nucleotide sugar metabolism, as well as starch and sucrose metabolism, were identified. These pathways may play a role in maintaining the plant’s sucrose/starch ratio, and can indirectly reflect trehalose metabolism activity, particularly through Trehalose-6-phosphate (Tre6P), a key signaling molecule linking sucrose availability and starch degradation. The defense response against infections was shaped by cultivar-specific pathways that might reflect unique defense mechanisms.

### 2.9. Genomic Variation Profiling

#### 2.9.1. Detection, Classification, and Functional Relevance of SNPs and InDels

**Figure 8:**
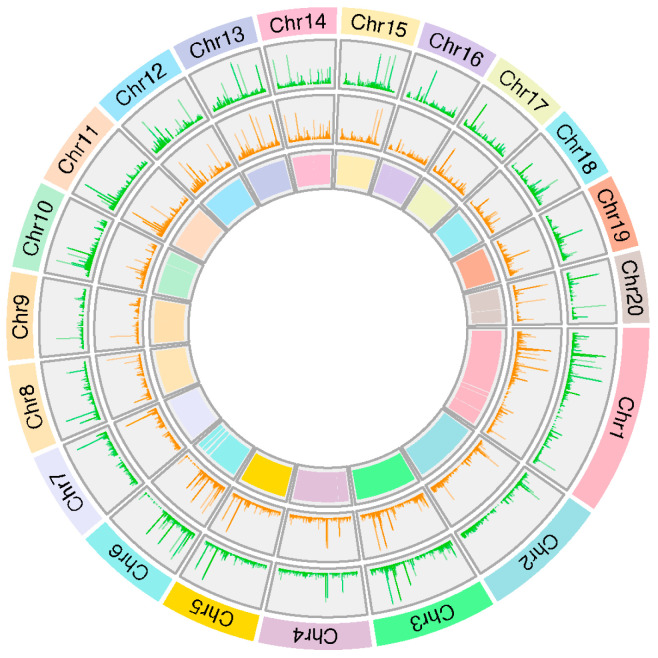
Chromosomal distribution of SNPs in *C. pepo* cultivars. Circular plot showing the SNPs distribution across the chromosomes of two *C. pepo* cultivars. From the outer to the inner ring: chromosome numbers (Chr1–Chr20), SNP density in 968Rb in green, SNP density in True French in orange, and gene distribution in the inner ring.

A transcriptome variant calling was performed to identify SNPs and InDels within the transcripts. This analysis was conducted comparing both genotypes, 968Rb (partial resistant) and TF (susceptible), against the reference genome of *C. pepo* (v4.1). A total of 9232 and 8451 high-quality homozygous SNPs were detected in 968Rb and TF, respectively, of which 7463 and 5525 were private to 968Rb and TF genotypes. In addition, a total of 7813 InDels were identified in the partially resistant genotype, of which 4060 were insertions and 3753 deletions, while 6616 InDels were detected in the susceptible genotype; detailed information on the number, impact, and functional class of SNPs and InDels is provided in Supplementary Table S6a–c. SNP distribution revealed a predominantly uniform pattern in both *C. pepo* cultivars (Figure 8).

**Figure 9:**
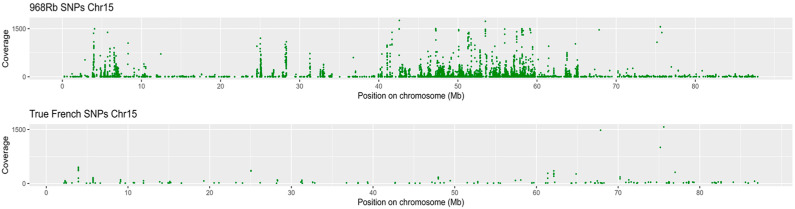
SNP density along chromosome 15 in *C. pepo* cultivars. SNPs density along chromosome 15 for the two cultivars: 968Rb on top and True French on bottom. Coverage values (y-axis) are plotted against chromosomal positions (x-axis in Mb).

Instead, a contrasting pattern was observed on chromosome 15 where 968Rb presented a higher SNP density compared to TF in the region from 3.7 Mb to 6.2 Mb (Figure 9). This region is of particular interest as it encompasses an SNP associated with the PR1 gene (Cp4.1LG15g04130) that has been identified as a central hub in the functional co-expression network connecting multiple biotic stress response proteins (Supplementary Figure S3 and Table S7).

Additionally, an SNP linked to a *GATA* gene (Cp4.1LG15g03070) was detected in this region, highlighting its putative involvement in the resistance process. The cultivar 968Rb exhibits specific regions with higher diversity, potentially linked to powdery mildew response. Therefore, these findings suggest that specific regions with contrasting SNP patterns may contribute to *C. pepo* response to *P. xanthii*. Variants were categorized into four groups based on their effect: high, moderate, modifier, and low (Supplementary Figure S4), and analyzed by GO term enrichment analysis (Supplementary Figures S5–S8). In 968Rb, the upstream region showed a strong enrichment in catabolic and metabolic processes, including allantoin and purine degradation, whereas True French was mainly associated with carbohydrate metabolism and protein regulation.

#### 2.9.2. Genetic Variation Within K-Means Expression Clusters

**Figure 10:**
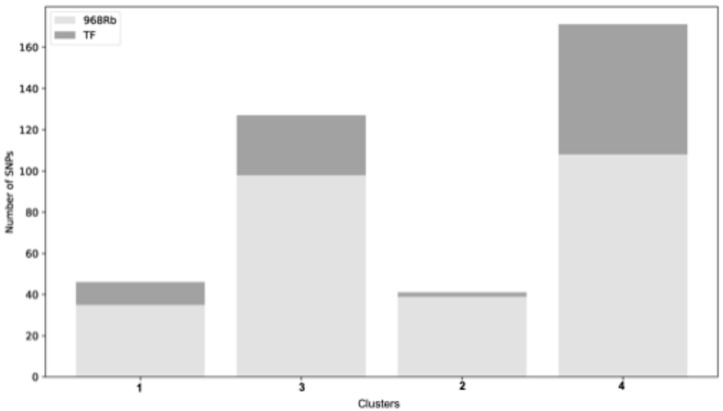
Comparison of SNP counts across K-means clusters. Comparison of SNPs identified in K-means clusters between the two *C. pepo* cultivars. The stacked bar plot displays the total number of SNPs in each cluster (1 to 4), with the light gray portion representing SNPs from 968Rb and the dark gray portion representing SNPs from TF.

The K-means clusters were investigated for the number of DEGs with SNPs and InDels. The goal was to investigate how the genetic variation was distributed across the clusters in the two cultivars. The results demonstrated that 968Rb exhibits a higher number of variants across all clusters when compared to TF (Figure 10). In Cluster 1, 968Rb contains 35 variants, while TF has only 11. Similarly, in Cluster 3, 968Rb has 98 variants, while TF has 29. In Cluster 4, 968Rb presents 108 variants, compared to 63 TF variants. Finally, in Cluster 2, 968Rb shows 39 variants, whereas TF has just 2. These results indicated that 968Rb displays a higher genetic diversity within each cluster. The higher number of variants in 968Rb, across all clusters, suggests that a wider range of genes can potentially contribute to a strong and effective response. To assess the relationship between genetic variability and gene expression response, the variant-to-gene ratio across the four K-means clusters was calculated. This ratio provides a way to compare how densely genetic variants are distributed across genes in different cultivars and clusters. Despite having a consistently lower number of DEGs across all clusters, the partially resistant cultivar 968Rb exhibits a significantly higher variant-to-gene ratio compared to TF. Specifically, in Cluster 1, 968Rb shows a ratio of 0.28, while TF reaches only 0.05; in Cluster 3, the ratio is 0.95 in 968Rb and 0.10 in TF; in Cluster 2, 968Rb has a ratio of 0.87, compared to 0.007 in TF; and in Cluster 4, 968Rb presents a ratio of 0.81, while TF shows a much lower value of 0.19. Fisher’s Exact Test was applied to compare the number of genes with and without variants across cultivars and clusters. The results confirmed that 968Rb has a significantly higher proportion of DEGs with variants in all clusters with a *p*-value < 0.01. These results indicate that, even though TF displays a larger transcriptomic response with more DEGs, the SNP density within DEGs is notably higher in 968Rb. This may reflect a targeted accumulation of genetic variation in the partially resistant genotype.

## 3. Discussion

Powdery mildew has spread throughout the world since its initial report, causing multiple yield losses in *Cucurbita* species. In this work, transcriptomic and genomic analyses were conducted to identify genes and pathways involved in the C. *pepo* defense mechanisms. The results obtained shed light on the molecular basis of the *C. pepo*–*P. xanthii* interaction in both partially resistant 968Rb and the susceptible True French cultivars. The partially resistant genotype 968Rb exhibited significantly lower infection rates, disease severity score, and leaf area coverage compared to the susceptible TF genotype. However, transcriptomic profiling revealed that the susceptible TF exhibited a larger number of DEGs than 968Rb, highlighting that a broad transcriptomic response is not necessarily indicative of effective resistance [11,22,23,24]. To better understand the complexity of the transcriptional responses and their genetic underpinnings, this study integrated conventional genomic analyses with AI-based methods, providing a framework to explore the functional organization of gene expression and the genetic basis of resistance. The algorithm K-means clustering showed that the Cluster 3 in 968Rb was enriched in cell wall remodeling genes. GPT-4 interpreted it as “cell wall reinforcement and metabolic adjustment”. The 968Rb upregulated genes, such as *Cp4.1LG01g09050*, *Cp4.1LG02g12520*, and *Cp4.1LG20g03720*, belong to the XTH (Xyloglucan endo-transglucosylase/hydrolase) family which is involved in maintaining cell wall integrity in response to PM [25,26,27,28]. GO enrichment analysis confirmed a significant overrepresentation of genes related to cell wall biogenesis in 968Rb. Among these, the genes involved in the biosynthesis of UDP-sugars, including UDP-xylose and UDP-arabinose, were found to be upregulated (e.g., *Cp4.1LG03g14330*, *Cp4.1LG14g02610*, *Cp4.1LG17g02910*). This finding supports a rapid synthesis and remodeling of the cell wall to establish an effective physical barrier to the pathogen [29]. In 968Rb, several extensin proteins were also upregulated, indicating an adaptive mechanism for maintaining cell wall integrity. The role of structural proteins such as extensins, proline-rich proteins, and glycine-rich proteins for cell wall resilience during infection has been shown to be crucial in resistant genotypes [30,31]. In contrast, a less coordinated response that could compromise the structural stability of the cell wall under pathogen attack was observed in TF. In addition, the upregulation of *Cp4.1LG19g00700*, a polygalacturonase-inhibiting protein (*PGIP*) in the resistant genotype, proved critical to inhibit PM polygalacturonases that degrade the plant cell wall [32]. The overexpression of *PGIP1s* also enhances resistance to *Fusarium graminearum* and *Rhizoctonia solani* in *rice* and *Arabidopsis*, respectively [33,34]. Taken together, these findings provided a clearer insight into the tightly regulated cell wall reinforcement complex implemented in the resistant genotype. The *fructose-bisphosphate aldolase* (*FBA*) genes, showing a pivotal role in plant stress response [35], resulted in being upregulated in 968Rb. Among them, it is worth noting that *Cp4.1LG02g00380*, identified in previous studies investigating stress-related pathways [36,37], was upregulated in 968Rb and downregulated in TF. Following *P. xanthii* infection, FBA genes interact with hormone signaling genes for activating specific pathways. In rice, FBA genes are known to play a role in signaling pathways related to resistance against both *Rhizoctonia solani* and brown planthopper [38,39]. AI-based clustering further supported its crucial role, identifying in 968Rb a transcriptional module (Cluster 4) enriched for genes associated with immune signal modulation and metabolic reprogramming, consistent with post-activation or downstream responses. GPT-4 interpreted this as a “general stress-adaptive signaling” cluster, reflecting a transcriptional response likely aimed at maintaining homeostasis. In addition, the cultivar 968Rb displayed a strong enrichment in porphyrin metabolism and ribosome-related pathways to adjust its immune response and keep its metabolism balanced [40,41]. Carbohydrate metabolism also appeared modulated, with alterations in the sucrose/starch ratio affecting trehalose levels. This sugar is known to influence hormonal signaling during stress and has been implicated in stress responses in cucurbits [42]. The upregulation of trehalose-related pathways in 968Rb may contribute to enhanced resilience as reported in other Cucurbits challenged by *Pseudomonas syringae* and osmotic stress [43,44]. Additionally, genes involved in allantoin catabolism showed enrichment in SNPs within regulatory regions in 968Rb. In melon, a gene functionally annotated as allantoate amidohydrolase (*Cmpmr2F*), converting the allantoate coming from allantoin to ureidoglycine, was identified as a pivotal player in the resistance to powdery mildew [45]. Allantoin can interact with jasmonic acid for inducing resistance responses to powdery mildew in melon [46]. All these results emphasize an intricate and synchronized metabolic adjustment in the partial resistant cultivar 968Rb, which facilitates both the activation of defense mechanisms and the preservation of cellular balance during stress. The combination of hormonal signaling, energy metabolism, and stress adaptation pathways seems crucial for preventing damage and utilizing energy efficiently, ultimately contributing to enhanced resistance to *P. xanthii.* The partially resistant 968Rb genotype displayed a significant upregulation of genes involved in early immune responses. AI-based K-means clustering identified a transcriptional module (Cluster 1) characterized by highly upregulated genes involved in early immune activation through pattern recognition signaling. This cluster includes genes associated with early immune perception and signal transduction, such as membrane-bound pattern recognition receptors (PRRs), kinases, and *WRKY/MYB* transcription factors typical of pattern-triggered immunity (PTI). The identification of this cluster supports the idea that the partially resistant cultivar mounts a coordinated early defense response involving these key signaling components. For instance, *Cp4.1LG06g05240*, a gene coding for a *leucine-rich repeat transmembrane protein kinase* (*LRR-RLK*), involved in the detection of pathogen-associated molecular patterns (PAMPs) [47,48,49] resulted in being upregulated in 968Rb and downregulated in TF. Similarly, the gene *Cp4.1LG15g04130*, encoding a PR1-like protein, was in the high-density SNP region on chromosome 15 and showed contrasting expression between 968Rb and TF. PR1 expression was significantly higher in 968Rb, in line with reports from resistant *C. sativus* and *C. moschata* under pathogen infection [50,51]. Previous studies have reported a higher proportion of unique SNPs on chromosome 10 in 968Rb, highlighting distinct genomic regions with notable variant densities in this cultivar [52]. Notably, an SNP was also detected in the nearby gene *Cp4.1LG15g03070*, encoding a *GATA* transcription factor, suggesting that this genomic region may play a coordinated role in resistance mechanisms. *GATA* transcription factors constitute a crucial gene family that regulates growth, development, and response to different biotic and abiotic stresses [53]. In other cucurbit species like *Cucumis melo* and *Cucumis sativus*, they have been implicated in resistance to pathogens like downy mildew and powdery mildew [54,55]. By contrast, in the susceptible cultivar TF, the chitin catabolic process, including various chitinase genes, was enhanced. Chitinases degrade chitin, a major component of fungal cell walls. Although TF shows induction of chitinase genes, this response might be ineffective since *P. xanthii* is known to evade chitin-triggered immunity and inhibit host chitinases [56]. Cucurbits are generally highly sensitive to chitin elicitation, which may paradoxically enhance susceptibility [57]. This pattern corresponds to Cluster 2 in TF, characterized by “Oxidative stress response and redox metabolism”, which is indicative of a general basal stress response lacking immune-specific transcriptional activation. Despite the general activation of stress pathways, phenylpropanoid biosynthesis was suppressed in TF, possibly weakening the synthesis of defense-related secondary metabolites. The enrichment of some related pathways, like the biosynthesis of secondary metabolites, plant hormone signal transduction, and MAPK signaling pathway, might support the synthesis of defense-related compounds for providing a quick response to pathogen attacks [58,59]. Overall, integrating AI-based clustering with traditional analyses highlighted how 968Rb predominantly activates early and targeted immune responses (Cluster 1), while TF mainly engages a general stress and redox response (Cluster 2), helping to explain the observed differences in resistance. These results also highlight the promise of hybrid approaches, in which conventional genomic analyses are integrated with AI-based strategies and biologically informed models. Such frameworks can improve interpretability, capture complex plant responses more accurately, and provide scalable tools for breeding programs [60]. These findings provide novel insights into the molecular basis of partial resistance to *P. xanthii* and offer a foundation for future functional validation and breeding applications.

## 4. Materials and Methods

### 4.1. Plant Materials and Podosphaera xanthii Inoculation

In this study, two genotypes of *C. pepo* were used: a partially resistant cultivar (968Rb) and a susceptible cultivar (TF) to *P. xanthii*. The seeds used in our study were kindly provided by Prof. Paris Harry (Department of Vegetable Crops, Agricultural Research Organization, Israel), who originally established and characterized them in his laboratory [18]. Fifty plants of each genotype, for both inoculated (i) and non-inoculated (ni) treatments, were grown in pots and maintained in a growth chamber under controlled temperature and humidity.

The fungal isolate used in this study was derived from local powdery mildew populations. After surface sterilization with HgCl_2_, infected plant material was transferred onto detached zucchini cotyledons cultured in vitro. Once fungal growth was visibly established, the pathogen was identified as *Podosphaera xanthii* through light microscopy. Single-spore cultures were subsequently obtained and maintained as a stable inoculum source on zucchini cotyledons, grown in vitro on agar medium at 25 ± 1 °C under a 16 h photoperiod. All inoculations were performed at a strictly defined and uniform plant developmental stage: seedlings aged 17–20 days, bearing two fully expanded true leaves [20], ensuring developmental synchrony across biological replicates. From these 50 plants, fully expanded leaves positioned at the same node and developmental level were collected for the detached-leaf assay. A total of 45 leaves from 968Rb and 38 leaves from TF were selected, depending on plant uniformity and leaf quality at the time of sampling. Detached leaves were placed in Petri dishes on moistened perlite and inoculated with *P. xanthii.* All sampled leaves were at the same physiological stage (full expansion), to reduce intra-sample variability and obtain infection-synchronized tissue suitable for transcriptomic analysis.

Disease assessments were initiated at 2 days post-inoculation (dpi) and continued weekly until susceptible plants exhibited complete leaf surface colonization. At each assessment, the number of infected leaves, the total number of leaves, the percentage of leaf area affected by disease symptoms, and a visual disease severity score on a 0–3 scale (0 = no visible symptoms; 3 = extensive powdery mildew coverage) were recorded. Statistical differences in disease severity between genotypes were evaluated using Duncan’s multiple range test.

### 4.2. Total RNA Extraction

Leaves were collected two days post-inoculation (dpi), when the first symptoms appeared, to study the gene expression changes during the early infection phase. Three biological replicates of frozen zucchini leaves were collected from both inoculated and not-inoculated samples to perform a total RNA extraction. According to Heidary M. et al. [61], the RNA purification was conducted using the TRIzol^®^ Reagent (Thermo Fisher Scientific, Waltham, MA, USA) and DNase I was used to eliminate any DNA contamination. The RNA samples were quantified using NanoDrop ND-1000 Spectrophotometer (Nano-Drop Technologies, Wilmington, DE, USA). Additionally, RNA integrity was checked by horizontal electrophoresis on an agarose gel stained with GelRed^®^ Nucleic Acid Stain 10,000X (Biotium Inc., Fremont, CA, USA) under UV light using a UV Gel Doc^TM^ system (Bio-Rad Laboratories, Hercules, CA, USA) and confirmed by Bioanalyzer (Agilent Technologies, Santa Clara, CA, USA). The latter was conducted to verify the integrity of the RNA samples and to ensure their accurate quantification prior to subsequent analyses.

### 4.3. Transcriptomic Sequencing, Mapping and Differential Expression Analysis

RNA samples were converted into cDNA using the QuantiTect Reverse Transcription kit (Qiagen, Hilden, Germany). Sequencing was carried out on the Illumina HiSeq 1500 platform via paired-end 100 base pair (bp) sequencing, utilizing a strand-specific library. The quality of the high-throughput sequencing data was assessed using FastQC v0.11.9 (Babraham Bioinformatics, Cambridge, UK) software [62]. Only high-quality reads with a quality score of 35 and a minimum length of 25 bp were selected for further downstream analysis. High-quality reads were aligned against the *C. pepo* reference genome (v4.1, PRJNA386743) [63,64].

Differential gene expression (DEG) analysis was conducted using the edge R package v3.32.1 within R v4.0.3 (R Core Team, Vienna, Austria) [65]. Genes were considered significantly differentially expressed if they met two criteria: a false discovery rate (FDR) of the statistical test less than 0.05 and an absolute fold change (|log2FC|) greater than or equal to zero (|log2FC| ≥ 0). Following differential expression analysis, four distinct DEG sets were identified: two representing upregulated genes and two representing downregulated genes in each cultivar. To visualize shared and unique DEGs among the four defined groups (968 upregulated, 968 downregulated, TF upregulated, and TF downregulated), an UpSet plot was generated by using the UpSetR package v1.4.0 in R [66].

### 4.4. Random Forest Classification of Variables

To identify the most relevant variables in the classification of DEGs between the two *C. pepo* varieties, Random Forest (RF) algorithm was utilized. This classifier was built by training multiple decision trees on random subsets of the data, with a configuration optimized to balance classification accuracy and computational efficiency. For classifying DEGs, several parameters of the differential analysis were used, including logFC (logarithm of fold change), logCPM (logarithm of counts per million), *p*-value, F-value, and FDR (false discovery rate). To determine which of these variables had the greatest influence on classification, the Feature Importance analysis computed with Random Forest Classifier from the scikit-learn library v1.3.2 (INRIA, Paris, France) in Python v3.10.6 was employed [67]. The Feature Importance was calculated by evaluating the Gini index *Gini* (*t*), as in Equation (1):

(1) $$Gini\left(t\right)=1-\sum_{i=1}^{C}p_{i}^{2}$$

where $p_{i}$ is the proportion of samples belonging to class i in node t and C is the number of possible classes. The higher the reduction in impurity, the more important the feature is considered for classification. To validate the importance of the features identified by the RF classifier, Permutation Feature Importance was calculated using the scikit-learn library’s permutation_importance function in Python [67].

### 4.5. Clustering Algorithms for DEGs Classification

To explore the underlying structure of the DEGs between these two *C. pepo* varieties, clustering was performed using three different algorithms: Agglomerative Clustering, K-means, and Self-Organizing Map (SOM). All three methods were trained using two key features derived from RF analysis: logFC and logCPM.

#### 4.5.1. K-Means

K clustering was performed using the scikit-learn library’s K-means module in Python [67]. K-means is based on the following objective function (*J*, Equation (2)), which is minimized during the clustering process:

(2) $$J={\sum_{i=1}^{n}\sum_{k=1}^{k}I\left(c_{i}=k\right)∥x_{i}-\mu_{k}∥}^{2}$$

where *J* is the objective function that K-means seeks to minimize, *n* represents the number of data points, *k* is the number of clusters, **I**$\left(c_{i}=k\right)$ is an indicator function that assigns the point $x_{i}$ to cluster *k*, $\mu_{k}$ is the centroid of cluster *k*, and $∥x_{i}-\mu_{k}∥$ is the squared Euclidean distance between the data point $x_{i}$ and the cluster centroid $\mu_{k}$. To choose the optimal number of clusters, k, the Silhouette Score was used to evaluate several configurations, from k = 2 to k = 9.

#### 4.5.2. Self-Organizing Map

A Self-Organizing Map (SOM) was implemented using the MiniSom library v2.3 in Python [68]. A 2 × 2 grid was selected for the SOM configuration, with a total of 5000 iterations applied to ensure a robust clustering and a sufficient stable model convergence. After the training, each gene was assigned to a cluster corresponding to the Best Matching Unit (BMU) in the grid, determined based on its proximity to the corresponding weight vector in the SOM. The BMU ($w\left(t+1\right))$ was calculated as follows (Equation (3)):

(3) $$w\left(t+1\right)=w\left(t\right)+\eta \left(t\right)\cdot h_{i}\left(t\right)\cdot (x-w\left(t\right))$$

where $w\left(t\right)$ represents the weight of the BMU at time t, $\eta \left(t\right)$ is the learning rate at time t, $h_{i}\left(t\right)$ is the proximity function for the BMU, which defines how strongly the BMU is influenced by the input vector x, and x is the input vector, representing DEG’s logFC and logCPM values.

#### 4.5.3. Agglomerative Clustering

The algorithm was implemented using the scikit-learn library’s Agglomerative Clustering module in Python [67]. Ward’s linkage method was employed in this study, which minimizes the sum of squared differences within all clusters. The formula for calculating the distance between the clusters ($d_{Ward}^{2}\left(A,B\right))$ is given by Equation (4):

(4) $$d_{Ward}^{2}\left(A,B\right)=\frac{n_{A}\cdot \;n_{B}}{n_{A}\;+\;n_{B}}∥\mu_{A}-\mu_{B}{∥}^{2}$$

where $d_{Ward}^{2}\left(A,B\right)$ is the distance between clusters A and B, $n_{A}$ and $n_{B}$ are the numbers of points in clusters A and B, $\mu_{A}$ and $\mu_{B}$ are the centroids of clusters A and B, and ${∥\mu_{A}-\mu_{B}∥}^{2}$ is the squared Euclidean distance between the centroids of the clusters.

Principal Component Analysis (PCA) was applied to reduce the dimensionality of the feature space for enabling the visualization of the clustering results, coming from the three clustering algorithms employed, in two dimensions. PCA provided a 2D scatter plot representation of the clusters, to visualize the distribution of genes across the two principal components. All the representations derived from these analyses were carried out using the Matplotlib v3.7.1 and Seaborn v0.12.2 libraries in Python [69]. The quality of the clustering methods was evaluated using various indices: Calinski–Harabasz Index (CHI), Davies–Bouldin Index (DBI), and Adjusted Rand Index (ARI). Each index offers a complementary perspective on the clustering performance and was performed using scikit-learn libraries on Python [67]. The results were used to assess the best performing clustering method in terms of both separation and consistency, thereby confirming the robustness of the clustering approach.

### 4.6. Conventional Functional and Orthology Annotation

Functional annotation was performed with MapMan v3.7.0 [70] using Mercator pipeline [71] to facilitate the assignment of genes to MapMan functional categories (bin). MapMan bin membership of individual genes provided a comprehensive overview of all differentially expressed genes in both genotypes. This, combined with the gene classification tool available on the Cucurbit Genomics Database (CuGenDB v.2) [72] and the literature review, enabled a highly detailed functional annotation.

In addition, a gene ontology (GO) enrichment analysis and KEGG pathways analysis were performed. The first one was conducted using ShinyGO web tool v0.76.3 [73] which applies Fisher’s Exact Test and Benjamini–Hochberg correction to determine statistically significant GO terms (FDR cutoff < 0.05). All three GO ontologies such as biological process, cellular component, and molecular function were analyzed. KEGG enrichment analysis was performed in two steps. The KEGG Automatic Annotation Server (KAAS v2.1) was first used [74] to assign KEGG Orthology (KO) terms to all DEGs. Subsequently, these KO terms were used within Kyoto Encyclopedia of Genes and Genomes [75] to identify enriched KEGG pathways. In this case, as in previous analyses, an FDR-corrected *p*-value threshold of less than or equal to 0.05 was used to determine the significance of enriched pathways in DEGs.

An orthology analysis for all genes was also performed to identify direct orthologs in *Arabidopsis thaliana* using The Arabidopsis Information Resource (TAIR) website [76]. A 3D protein–protein interaction network with STRING database v12.0 [77] was built using *A. thaliana* orthologs proteins to highlight crucial key genes interactions.

### 4.7. AI-Based Biological Interpretation via GPT-4

The genes belonging to K-means clusters were functionally characterized using a multi-step workflow combining traditional gene ontology (GO) enrichment and AI-based interpretation. All DEGs were annotated using EggNOG-mapper v2 [78] which assigns GO terms to each gene based on orthology to functionally characterized proteins. GO terms belonging to the three ontology domains were extracted and mapped to the clustered DEGs. For each cluster and for each cultivar, the most frequent GO terms were selected. To explain the biological significance of the top GO terms for each cluster, a natural language generation approach was adopted which was inspired by Hu et al. [79]. GPT-4 (OpenAI) was used to generate the biological explanation from each GO term set and submitted via API to use a standardized prompt designed to produce consistent and reproducible output. For each cluster, the model was asked to propose a clear, descriptive title and outline the key biological, molecular, and cellular processes involved, and it also suggested the most likely gene types. The outputs were then compared with conventional GO enrichment results to evaluate consistency and improve the interpretability of cluster-specific functions.

### 4.8. Variant Calling: SNPs and InDel Investigation

The variant analysis was conducted to detect single nucleotide polymorphism (SNP) and the insertions/deletions (InDel) by aligning the 968Rb and TF transcript sequences against the *C. pepo* reference genome (v 4.1) [64]. The analysis was performed on BAM files, generated from susceptible and partial resistance genotypes using BCFtools [80]. Different quality filters were applied, retaining variants with allele frequency (AF) > 0.75, minimum quality (QUAL) and minimum genotype quality (GQ) equal to 30, read depth (DP) between 5 and 110, and minimum mapping quality (MQ) ≥ 20. Variants were functionally annotated using SnpEff [81] which predicted their effects and classified them into four levels of impact: high, low, moderate, and modifier. Subsequently, variants were also annotated according to their genomic location, including upstream regions (1–5 kb upstream of transcription start sites), downstream regions (1–5 kb downstream of transcription termination sites), introns, exons, and intergenic regions. Genes associated with upstream and downstream variants were identified using BEDTools (v2.30.0) [82] and functional enrichment analysis of these genes was performed using ShinyGO, as previously described.

To visualize the genomic distribution of SNPs and InDel across the 20 *C. pepo* chromosomes, a circos plot was generated using the circlize R package v0.4.15 [83]. Chromosomal lengths were defined based on the *C. pepo* reference genome (v4.1). SNP density tracks were plotted for both 968Rb and TF, allowing a direct comparison of their genomic variant distributions.

### 4.9. Genetic Variant Analysis Within DEG Clusters

To integrate gene expression results with genetic diversity analysis, a Python pipeline was developed to map gene variants and to link them to the DEG clusters identified by K-means. Annotation was performed by parsing the GFF file of the *C. pepo* reference genome (v4.1) to extract coordinates and IDs. The variants were then mapped to these genes and then merged with the K-means clustering results using the panda’s library. For each cluster, a binary matrix was constructed to indicate the presence or absence of variants, and the variant-to-gene ratio was calculated to have the density of variants relative to the number of DEGs. Finally, Fisher’s Exact Test was applied to verify the significance of the differences between the cultivars (*p* < 0.01).

## 5. Conclusions

This research combines conventional omics analysis with AI-based approaches to uncover the molecular basis of resistance to *P. xanthii* in *C. pepo*. AI-based clustering and functional annotation uncovered insights into unique defense strategies, highlighting coordinated gene modules and regulatory variants within the partially resistant cultivar. The 968Rb genotype exhibited a tightly regulated defense strategy, characterized by transcriptional modules associated with early immune signaling, metabolic reprogramming, and cell wall remodeling. The presence of high-impact variants in the regulatory regions of key defense genes supports the hypothesis of an adaptive transcriptional process, enabling a more rapid and efficient response to fungal attacks. This integrative framework highlights the power of AI-enhanced functional genomics in decoding complex resistance mechanisms. These findings pave the way for breeding strategies, offering a more precise and sustainable approach to improving disease resistance.

## Figures and Tables

**Table 1:** Clustering quality evaluation indices. This table presents the results of the Adjusted Rand Index, Calinski–Harabasz Index, and Davies–Bouldin Index used to assess the quality of the clustering results.

Algorithms	Adjusted Rand Index	Calinski–Harabasz Index	Davies–Bouldin Index
agglomerative clustering	0.5037	1060.4	0.9368
k-means	0.4142	1171.0	0.8430
self-organizing maps	0.5491	1126.4	0.9082

**Table 2:** Cluster-specific biological functions characterized through GO enrichment and GPT-4 interpretation. Each row reports a K-means cluster characterized by its expression trend, cultivar-specific origin, and a concise functional interpretation generated by GPT-4. Interpretations were generated by GPT-4 based on significantly enriched GO terms (FDR < 0.05), highlighting key processes such as immune signaling, cell wall remodeling, or oxidative stress response. This compact overview complements the complete GO term enrichment data available in Supplementary Table S5.

Cluster	Expression Pattern	Cultivar	GPT-4 Title	GPT-4 Biological Interpretation
**1**	Highly upregulated	968Rb	Early immune activation through pattern recognition signaling	Genes involved in early immune perception and signal transduction, activating membrane-bound PRRs, kinases, and *WRKY/MYB* transcription factors typical of PTI.
		True French	Transcriptional and hormonal regulation in stress signaling	Genes involved in general stress-responsive regulation, including hormonal crosstalk and transcriptional activation under abiotic and biotic stimuli; less immune-specific.
**2**	Highly downregulated	968Rb	Repression of growth- and homeostasis-related pathways	Strong suppression of developmental, transport, and hormonal response genes, indicating metabolic shift prioritizing defense overgrowth and nutrient flow.
		True French	Oxidative stress response and redox metabolism	Enrichment in redox and detoxification pathways, including ROS scavenging; general basal stress response without activation of immune-specific transcription factors.
**3**	Moderately upregulated	968Rb	Cell wall reinforcement and metabolic adjustment	Genes involved in structural defense via cell wall remodeling and protein synthesis; also linked to chloroplast activity and metabolic adaptation.
		True French	Membrane-linked signaling and transcriptional regulation	General stress-adaptive signaling and transcriptional activation, likely associated with hormonal adjustment and metabolic regulation.
**4**	Moderately downregulated	968Rb	Late-stage modulation of immune response	Fine-tuning of immune signaling and transport activity during late immune response phase; includes proteases and hormone modulators.
		True French	Signal transduction and basal metabolic activity	Metabolic and signaling adjustment under stress, lacking strong immune-specific functional signatures.

## Data Availability

Raw data have been deposited in Figshare under https://figshare.com/s/b5b83de8622d9ec5975a accessed on 24 November 2025. The data are currently private and accessible to reviewers via a shared link. They will be made publicly available upon publication.

## Supplementary Materials

The following supporting information can be downloaded at https://www.mdpi.com/article/10.3390/ijms262311488/s1.

## Abbreviations

The following abbreviations are used in this manuscript:

ABA	Abscisic Acid
AF	Allele Frequency
AI	Artificial Intelligence
ARI	Adjusted Rand Index
CHI	Calinski–Harabasz Index
CSL	Cellulose Synthase-Like
DBI	Davies–Bouldin Index
DEG(s)	Differentially Expressed Gene(s)
DL	Deep Learning
dpi	Days Post-Inoculation
DP	Read Depth (coverage)
ERF	Ethylene Response Factor
F	F-value
FBA	Fructose-Bisphosphate Aldolase
FDR	False Discovery Rate
GAE	UDP-D-Glucuronate 4-Epimerase
GATA	GATA Transcription Factor
GO	Gene Ontology
GQ	Genotype Quality
JA	Jasmonic Acid
KEGG	Kyoto Encyclopedia of Genes and Genomes
KO	KEGG Orthology
logCPM	Log Counts Per Million
logFC	Log Fold Change
MAPK	Mitogen-Activated Protein Kinase
ML	Machine Learning
MQ	Mapping Quality
NLR	Nucleotide-binding Leucine-rich Repeat protein
PCA	Principal Component Analysis
PGIP	Polygalacturonase-Inhibiting Protein
PM	Powdery Mildew
PPI	Protein–Protein Interaction
PR1	Pathogenesis-Related Protein 1
PRR(s)	Pattern Recognition Receptor(s)
PTI	Pattern-Triggered Immunity
RF	Random Forest
SA	Salicylic Acid
SNP(s)	Single Nucleotide Polymorphism(s)
SOM	Self-Organizing Map
Tre6P	Trehalose-6-Phosphate
TF	True French
UGE	UDP-D-Glucuronate 4-Epimerase
UXS	UDP-Glucuronic Acid Decarboxylase
XTH(s)	Xyloglucan Endotransglucosylase/Hydrolase(s)

## References

[1] FAO (2022). FAOSTAT Database. Food and Agriculture Organization of the United Nations. https://www.fao.org/faostat/en/#home.

[2] Andolfo G., Sánchez C.S., Cañizares J., Pico M.B., Ercolano M.R. (2021). Large-scale gene gains and losses molded the NLR defense arsenal during the Cucurbita evolution. Planta.

[3] Qu S.-P., Yang D., Yu H.-Y., Chen F.-Y., Wang K.-X., Ding W.-Q., Xu W.-L., Wang Y.-L. (2022). QTL analysis of early flowering of female flowers in zucchini (*Cucurbita pepo* L.). J. Integr. Agric..

[4] Xu X., Liu X., Yan Y., Wang W., Gebretsadik K., Qi X., Xu Q., Chen X. (2019). Comparative proteomic analysis of cucumber powdery mildew resistance between a single-segment substitution line and its recurrent parent. Hortic. Res..

[5] Cohen R., Burger Y., Katzir N. (2004). Monitoring physiological races of *Podosphaera xanthii* (syn. *Sphaerotheca fuliginea*), the causal agent of powdery mildew in cucurbits: Factors affecting race identification and the importance for research and commerce. Phytoparasitica.

[6] Miazzi M., Laguardia C., Faretra F. (2011). Variation in *Podosphaera xanthii* on cucurbits in Southern Italy. J. Phytopathol..

[7] Zhu Q., Wu C., Amanullah S., Liu S., Gao P., Wang X., Ma H., Zhu Z., Luan F., Kozik E.U., Paris H.S. (2016). Genome-Wide Association Study of Powdery Mildew Resistance in a Worldwide Collection of Melon (*Cucumis melo* L.) Germplasm. Cucurbitaceae 2016, XIth Eucarpia Meeting on Cucurbit Genetics & Breeding.

[8] Alavilli H., Lee J.-J., You C.-R., Poli Y., Kim H.-J., Jain A., Song K. (2022). GWAS Reveals a Novel Candidate Gene CmoAP2/ERF in Pumpkin (*Cucurbita moschata*) Involved in Resistance to Powdery Mildew. Int. J. Mol. Sci..

[9] Zhang P., Zhu Y., Zhou S. (2021). Comparative analysis of powdery mildew resistant and susceptible cultivated cucumber (*Cucumis sativus* L.) varieties to reveal the metabolic responses to *Sphaerotheca fuliginea* infection. BMC Plant Biol..

[10] Cao Y., Diao Q., Lu S., Zhang Y., Yao D. (2022). Comparative transcriptomic analysis of powdery mildew resistant and susceptible melon inbred lines to identify the genes involved in the response to *Podosphaera xanthii* infection. Sci. Hortic..

[11] Meng X., Yu Y., Song T., Yu Y., Cui N., Ma Z., Chen L., Fan H. (2022). Transcriptome Sequence Analysis of the Defense Responses of Resistant and Susceptible Cucumber Strains to *Podosphaera xanthii*. Front. Plant Sci..

[12] Lin E., Lane H.-Y. (2017). Machine learning and systems genomics approaches for multi-omics data. Biomark. Res..

[13] Guo X., Han J., Song Y., Yin Z., Liu S., Shang X. (2022). Using expression quantitative trait loci data and graph-embedded neural networks to uncover genotype–phenotype interactions. Front. Genet..

[14] Xu C., Jackson S.A. (2019). Machine learning and complex biological data. Genome Biol..

[15] Sharieff N.A.A., Sameer N.R. (2023). Artificial intelligence Techniques in Bioinformatics: Unravelling complex biological systems. Int. J. Adv. Res. Sci. Commun. Technol..

[16] Tong K., Chen X., Yan S., Dai L., Liao Y., Li Z., Wang T. (2024). PlantMine: A Machine-Learning Framework to Detect Core SNPs in Rice Genomics. Genes.

[17] Rezayi S., Kalhori S.R.N., Saeedi S. (2022). Effectiveness of Artificial intelligence for Personalized Medicine in Neoplasms: A Systematic review. BioMed Res. Int..

[18] Singh D., Sachdeva D., Singh L. (2025). Advancing breast cancer drug delivery: The transformative potential of bioinformatics and artificial intelligence. Curr. Cancer Ther. Rev..

[19] Vidanagamachchi S.M., Waidyarathna K.M.G.T.R. (2024). Opportunities, challenges and future perspectives of using bioinformatics and artificial intelligence techniques on tropical disease identification using omics data. Front. Digit. Health.

[20] Cohen R., Hanan A., Paris H. (2003). Single-gene resistance to powdery mildew in zucchini squash (*Cucurbita pepo*). Euphytica.

[21] Luo J., Xia W., Cao P., Xiao Z., Zhang Y., Liu M., Zhan C., Wang N. (2019). Integrated Transcriptome Analysis Reveals Plant Hormones Jasmonic Acid and Salicylic Acid Coordinate Growth and Defense Responses upon Fungal Infection in Poplar. Biomolecules.

[22] Kiani M., Szczepaniec A. (2018). Effects of sugarcane aphid herbivory on transcriptional responses of resistant and susceptible sorghum. BMC Genom..

[23] Kiani M., Bryan B., Rush C., Szczepaniec A. (2021). Transcriptional responses of resistant and susceptible wheat exposed to wheat curl mite. Int. J. Mol. Sci..

[24] De Mello U.S., Vidigal P.M.P., Vital C.E., Tomaz A.C., De Figueiredo M., Peternelli L.A., Barbosa M.H.P. (2020). An overview of the transcriptional responses of two tolerant and susceptible sugarcane cultivars to borer (*Diatraea saccharalis*) infestation. Funct. Integr. Genom..

[25] Cheng Z., Zhang X., Yao W., Gao Y., Zhao K., Guo Q., Zhou B., Jiang T. (2021). Genome-wide identification and expression analysis of the xyloglucan endotransglucosylase/hydrolase gene family in poplar. BMC Genom..

[26] De Caroli M., Manno E., Piro G., Lenucci M.S. (2021). Ride to cell wall: *Arabidopsis XTH11*, *XTH29* and *XTH33* exhibit different secretion pathways and responses to heat and drought stress. Plant J..

[27] Han J., Liu Y., Shen Y., Li W. (2023). A surprising diversity of Xyloglucan Endotransglucosylase/Hydrolase in wheat: New in sight to the roles in drought tolerance. Int. J. Mol. Sci..

[28] Qiao T., Zhang L., Yu Y., Pang Y., Tang X., Wang X., Li L., Li B., Sun Q. (2022). Identification and expression analysis of xyloglucan endotransglucosylase/hydrolase (XTH) family in grapevine (*Vitis vinifera* L.). PeerJ.

[29] Liu T., Shen C., Wang Y., Huang C., Shi J. (2014). New Insights into Regulation of Proteome and Polysaccharide in Cell Wall of *Elsholtzia splendens* in Response to Copper Stress. PLoS ONE.

[30] Sun Q. (2021). Structural variation and polysaccharide profiling of intervessel pit membranes. bioRxiv.

[31] Chen L., Xu S., Liu Y., Zu Y., Zhang F., Du L., Chen J., Li L., Wang K., Wang Y. (2022). Identification of key gene networks controlling polysaccharide accumulation in different tissues of *Polygonatum cyrtonema* Hua by integrating metabolic phenotypes and gene expression profiles. Front. Plant Sci..

[32] Cheng S., Li R., Lin L., Shi H., Liu X., Yu C. (2021). Recent Advances in Understanding the Function of the PGIP Gene and the Research of Its Proteins for the Disease Resistance of Plants. Appl. Sci..

[33] Liu N., Zhang X., Sun Y., Wang P., Li X., Pei Y., Li F., Hou Y. (2017). Molecular evidence for the involvement of a polygalacturonase-inhibiting protein, *GhPGIP1*, in enhanced resistance to *Verticillium* and *Fusarium* wilts in cotton. Sci. Rep..

[34] Wang R., Lu L., Pan X., Hu Z., Ling F., Yan Y., Liu Y., Lin Y. (2014). Functional analysis of *OsPGIP1* in rice sheath blight resistance. Plant Mol. Biol..

[35] Lu W., Tang X., Huo Y., Xu R., Qi S., Huang J., Zheng C., Wu C.-A. (2012). Identification and characterization of fructose 1,6-bisphosphate aldolase genes in *Arabidopsis* reveal a gene family with diverse responses to abiotic stresses. Gene.

[36] Sun H., Dai H., Wang X., Wang G. (2016). Physiological and proteomic analysis of selenium-mediated tolerance to Cd stress in cucumber (*Cucumis Sativus* L.). Ecotoxicol. Environ. Saf..

[37] Uçar S., Alım Ş., Kasapoğlu A.G., Yigider E., İlhan E., Turan M., Polat A., Dikbaş N., Aydın M. (2024). Genome-Wide Analysis and Characterization of FBA (Fructose 1,6-bisphosphate aldolase) Gene Family of *Phaseolus vulgaris* L.. J. Agric. Prod..

[38] Shinde S., Abida P.S., Saakre M., Bhaskar H., Beena R., Preetha R. (2024). Identification and comparative analysis of differential proteins expression in rice under biotic stress by protein sequencing. Cereal Res. Commun..

[39] Mutuku J.M., Nose A. (2012). Changes in the Contents of Metabolites and Enzyme Activities in Rice Plants Responding to *Rhizoctonia solani* Kuhn Infection: Activation of Glycolysis and Connection to Phenylpropanoid Pathway. Plant Cell Physiol..

[40] Liu J., Wang B., Li Y., Huang L., Zhang Q., Zhu H., Wen Q. (2020). RNA sequencing analysis of low temperature and low light intensity-responsive transcriptomes of zucchini (*Cucurbita pepo* L.). Sci. Hortic..

[41] Chen B.-H., Guo W.-L., Yang H.-L., Li Q.-F., Zhou J.-G., Li X.-Z. (2020). Photosynthetic properties and biochemical metabolism of *Cucurbita moschata* genotypes following infection with powdery mildew. J. Plant Pathol..

[42] Hassan M.U., Nawaz M., Shah A.N., Raza A., Barbanti L., Skalicky M., Hashem M., Brestic M., Pandey S., Alamri S. (2022). Trehalose: A key player in plant growth regulation and tolerance to abiotic stresses. J. Plant Growth Regul..

[43] Kopczewski T., Kuźniak E., Ciereszko I., Kornaś A. (2022). Alterations in Primary Carbon Metabolism in Cucumber Infected with *Pseudomonas syringae* pv *lachrymans*: Local and Systemic Responses. Int. J. Mol. Sci..

[44] Zhu F., Li M., Sun M., Jiang X., Qiao F. (2022). Plant hormone signals regulate trehalose accumulation against osmotic stress in watermelon cells. Protoplasma.

[45] Zhang T., Cui H., Luan F., Liu H., Ding Z., Amanullah S., Zhang M., Ma T., Gao P. (2023). A recessive gene *Cmpmr2F* confers powdery mildew resistance in melon (*Cucumis melo* L.). Theor. Appl. Genet..

[46] Dun B., Zhuo D., Shuai W., Yane S., Yahang L., Haonan C. (2023). Allantoin and jasmonic acid synergistically induce resistance response to powdery mildew in melon as revealed by combined hormone and transcriptome analysis. Sci. Hortic..

[47] Li B., Meng X., Shan L., He P. (2016). Transcriptional regulation of Pattern-Triggered immunity in plants. Cell Host Microbe.

[48] Yu X., Feng B., He P., Shan L. (2017). From Chaos to Harmony: Responses and Signaling upon Microbial Pattern Recognition. Annu. Rev. Phytopathol..

[49] Ma Q., Hu Z., Mao Z., Mei Y., Feng S., Shi K. (2022). The novel leucine-rich repeat receptor-like kinase MRK1 regulates resistance to multiple stresses in tomato. Hortic. Res..

[50] Hashemi L., Golparvar A.R., Nasr-Esfahani M., Golabadi M. (2020). Expression analysis of defense-related genes in cucumber (*Cucumis sativus* L.) against *Phytophthora melonis*. Mol. Biol. Rep..

[51] Guo W.-L., Yang H.-L., Zhao J.-P., Bian S.-J., Guo Y.-Y., Chen X.-J., Li X.-Z. (2023). A pathogenesis-related protein 1 of *Cucurbita moschata* responds to powdery mildew infection. Front. Genet..

[52] Perez-Moro C., D’Esposito D., Pérez-De-Castro A., Ercolano M., Capuozzo C., Guadagno A. (2024). Comparative analysis of *Cucurbita pepo* genomes shed light in agronomic traits variation. Res. Sq..

[53] Duan X., Yuan Y., Real N., Tang M., Ren J., Wei J., Liu B., Zhang X. (2024). Fine mapping and identification of candidate genes associated with powdery mildew resistance in melon (*Cucumis melo* L.). Hortic. Res..

[54] Zhang K., Jia L., Yang D., Hu Y., Njogu M.K., Wang P., Lu X., Yan C. (2021). Genome-Wide Identification, Phylogenetic and Expression Pattern Analysis of GATA Family Genes in Cucumber *(Cucumis sativus* L.). Plants.

[55] Zheng L., Tang L., Li J. (2024). Genome-wide identification of the GATA gene family in melon (*Cucumis melo*) and analysis of their expression characteristics under biotic and abiotic stresses. Front. Plant Sci..

[56] Martínez-Cruz J.M., Polonio Á., Ruiz-Jiménez L., Vielba-Fernández A., Hierrezuelo J., Romero D., De Vicente A., Fernández-Ortuño D., Pérez-García A. (2022). Suppression of Chitin-Triggered Immunity by a New Fungal Chitin-Binding Effector Resulting from Alternative Splicing of a Chitin Deacetylase Gene. J. Fungi.

[57] Polonio Á., Fernández-Ortuño D., De Vicente A., Pérez-García A. (2021). A haustorial-expressed lytic polysaccharide monooxygenase from the cucurbit powdery mildew pathogen *Podosphaera xanthii* contributes to the suppression of chitin-triggered immunity. Mol. Plant Pathol..

[58] Figueroa-Macías J.P., García Y.C., Núñez M., Díaz K., Olea A.F., Espinoza L. (2021). Plant Growth-Defense Trade-Offs: Molecular processes leading to physiological changes. Int. J. Mol. Sci..

[59] Abdelkhalek A., Király L., Al-Mansori A.-N.A., Younes H.A., Zeid A., Elsharkawy M.M., Behiry S.I. (2022). Defense Responses and Metabolic Changes Involving Phenylpropanoid Pathway and PR Genes in Squash (*Cucurbita pepo* L.) following *Cucumber mosaic virus* Infection. Plants.

[60] Dublino R., Ercolano M. (2025). Artificial intelligence redefines agricultural genetics by unlocking the enigma of genomic complexity. Crop. J..

[61] Heidary M., Pahlevan Kakhki M. (2014). TRIzol-Based RNA Extraction: A Reliable Method for Gene Expression Studies. J. Sci. Islam. Repub. Iran.

[62] Andrews S. (2010). FastQC: A Quality Control Tool for High Throughput Sequence Data. https://www.bioinformatics.babraham.ac.uk/projects/fastqc/.

[63] Zheng Y., Wu S., Bai Y., Sun H., Jiao C., Guo S., Zhao K., Blanca J., Zhang Z., Huang S. (2018). Cucurbit Genomics Database (CuGenDB): A central portal for comparative and functional genomics of cucurbit crops. Nucleic Acids Res..

[64] Montero-Pau J., Blanca J., Bombarely A., Ziarsolo P., Esteras C., Martí-Gómez C., Ferriol M., Gómez P., Jamilena M., Mueller L. (2017). De novo assembly of the zucchini genome reveals a whole-genome duplication associated with the origin of the *Cucurbita* genus. Plant Biotechnol. J..

[65] Robinson M.D., McCarthy D.J., Smyth G.K. (2009). edgeR: A Bioconductor package for differential expression analysis of digital gene expression data. Bioinformatics.

[66] Conway J.R., Lex A., Gehlenborg N. (2017). UpSetR: An R package for the visualization of intersecting sets and their properties. Bioinformatics.

[67] Pedregosa F., Varoquaux G., Gramfort A., Michel V., Thirion B., Grisel O., Blondel M., Prettenhofer P., Weiss R., Dubourg V. (2011). Scikit-Learn: Machine Learning in Python. J. Mach. Learn. Res..

[68] Vettigli G. (2018). MiniSom: Minimalistic and Numpy-Based Implementation of the Self Organizing Map [Computer Software]. GitHub. https://github.com/JustGlowing/minisom.

[69] Caswell T.A., Droettboom M., Lee A., Hunter J., Firing E., Sales De Andrade E., Hoffmann T., Stansby D., Klymak J., Varoquaux N. (2020). Matplotlib/Matplotlib: REL: v3.3.0. https://github.com/matplotlib/matplotlib/tree/v3.3.0.

[70] Thimm O., Bläsing O., Gibon Y., Nagel A., Meyer S., Krüger P., Selbig J., Müller L.A., Rhee S.Y., Stitt M. (2004). mapman: A user-driven tool to display genomics data sets onto diagrams of metabolic pathways and other biological processes. Plant J..

[71] Lohse M., Nagel A., Herter T., May P., Schroda M., Zrenner R., Tohge T., Fernie A.R., Stitt M., Usadel B. (2013). Mercator: A fast and simple web server for genome scale functional annotation of plant sequence data. Plant Cell Environ..

[72] Yu J., Wu S., Sun H., Wang X., Tang X., Guo S., Zhang Z., Huang S., Xu Y., Weng Y. (2022). CuGenDBv2: An updated database for cucurbit genomics. Nucleic Acids Res..

[73] Ge S.X., Jung D., Yao R. (2019). ShinyGO: A graphical gene-set enrichment tool for animals and plants. Bioinformatics.

[74] Moriya Y., Itoh M., Okuda S., Yoshizawa A.C., Kanehisa M. (2007). KAAS: An automatic genome annotation and pathway reconstruction server. Nucleic Acids Res..

[75] Kanehisa M., Furumichi M., Sato Y., Kawashima M., Ishiguro-Watanabe M. (2022). KEGG for taxonomy-based analysis of pathways and genomes. Nucleic Acids Res..

[76] TAIR (2000). The Arabidopsis Information Resource. https://www.arabidopsis.org/aboutarabidopsis.html.

[77] Szklarczyk D., Kirsch R., Koutrouli M., Nastou K., Mehryary F., Hachilif R., Gable A.L., Fang T., Doncheva N.T., Pyysalo S. (2022). The STRING database in 2023: Protein–protein association networks and functional enrichment analyses for any sequenced genome of interest. Nucleic Acids Res..

[78] Cantalapiedra C.P., Hernández-Plaza A., Letunic I., Bork P., Huerta-Cepas J. (2021). eggNOG-mapper v2: Functional Annotation, Orthology Assignments, and Domain Prediction at the Metagenomic Scale. Mol. Biol. Evol..

[79] Hu M., Alkhairy S., Lee I., Pillich R.T., Fong D., Smith K., Bachelder R., Ideker T., Pratt D. (2025). Evaluation of large language models for discovery of gene set function. Nat. Methods.

[80] Li H., Handsaker B., Wysoker A., Fennell T., Ruan J., Homer N., Marth G., Abecasis G., Durbin R. (2009). The Sequence Alignment/Map format and SAMtools. Bioinformatics.

[81] Cingolani P. (2012). Variant annotation and Functional Prediction: SNPEFF. Methods in Molecular Biology.

[82] Quinlan A.R., Hall I.M. (2010). BEDTools: A flexible suite of utilities for comparing genomic features. Bioinformatics.

[83] Gu Z., Gu L., Eils R., Schlesner M., Brors B. (2014). *circlize* implements and enhances circular visualization in R. Bioinformatics.

