# Supplementary material for: AI-Integrated Omics Analysis Reveals Cultivar-Specific Resistance Mechanisms to Powdery Mildew in Cucurbita pepo
Source: Int J Mol Sci. 2025 Nov 27;26(23):11488. doi: 10.3390/ijms262311488 (PMC12692380; doi:10.3390/ijms262311488)
Supplement: Supplementary file 1 [file ijms-26-11488-s001.zip › Supplementary_figures_tables_descriptions.pdf]

SUPPLEMENTARY FIGURES AND TABLES DESCRIPTIONS

Supplementary Table S1. Summary of RNA-seq libraries from *C. pepo* genotypes 968Rb and True French (TF), inoculated and mock-treated. This table summarizes the RNA sequencing data for 12 libraries derived from the partially resistant genotype 968Rb and the susceptible genotype TF. Each genotype was analyzed under two conditions: inoculated with *P. xanthii* and control (mock-treated). For each sample, the number of paired-end reads is reported.

Supplementary Table S2. Differentially expressed genes in 968Rb (filtered by FDR < 0.05). This sheet lists the DEGs identified in the 968Rb genotype, filtered using an FDR threshold of 0.05. For each gene, the log fold change (logFC), false discovery rate (FDR), and other expression statistics are provided.

Supplementary Table S3. Differentially expressed genes in True French (TF) , filtered by FDR < 0.05. This sheet lists the DEGs identified in the True French genotype, filtered using an FDR threshold of 0.05. For each gene, the log fold change (logFC), false discovery rate (FDR), and other expression statistics are provided.

Supplementary Table S4. K-means clustering of DEG. K-means clustering results for differentially expressed genes (DEGs). The file includes gene IDs, assigned cluster (1 to 4), and associated expression values.

Supplementary Table S5. Enriched GO terms and AI interpretations per cluster and cultivar. Summary of enriched GO terms across clusters for both cultivars (968Rb and True French), with indication of expression pattern, cluster assignment, and AI-based biological interpretations generated with GPT-4.

Supplementary Table S5. Summary of genetic variants in *C. pepo* genotypes 968Rb and True French (TF).
S5a. Number of variants by type (SNPs, insertions, deletions).
S5b. Classification of variant effects by predicted impact (high, moderate, low, modifier).
S5c. Classification of variant effects by functional class (missense, nonsense, silent).

Supplementary Table S7. Conserved orthologous defense-related genes between *A. thaliana* and *C. pepo*. The table lists defense-related orthologous proteins from *A. thaliana* and their corresponding gene IDs in *C. pepo*, identified via sequence homology. The last column reports the transcriptional response in the two genotypes upon *P. xanthii* infection.

Supplementary Figure S1. Untreated leaf phenotype in resistant (968Rb) and susceptible (TF) genotypes. Detached leaves from *C. pepo* cultivars 968Rb and TF were incubated under sterile conditions without *P. xanthii* inoculation. Both leaves show healthy, turgid tissue with no visible signs of damage, serving as the baseline control to assess phenotypic differences upon pathogen challenge.

Supplementary Figure S2. Phenotypic response to *P. xanthii* infection at 3 days post-inoculation. Representative leaves from 968Rb and True French (TF) were collected 3 days after inoculation with *P. xanthii*. The susceptible TF cultivar shows extensive white powdery mildew coverage and chlorosis, while the resistant 968Rb displays limited fungal growth and preserved green tissue, highlighting differential resistance mechanisms.

Supplementary Figure S3. Protein–protein interaction (PPI) network of selected DEGs. PPI network based on *A. thaliana* orthologs of *C. pepo* DEGs. Nodes represent proteins and are colored according to cluster assignment: Cluster 1 (red), Cluster 2 (yellow), Cluster 3 (green), Cluster 4 (blue).

Supplementary Figure S4. Variant effect classification in DEGs. Donut charts summarize the classification of variant effects by predicted impact (high, low, modarate and modifier).

Supplementary Figure S5. GO term enrichment of upstream regions in 968Rb. Bar polar plot showing Gene Ontology enrichment of upstream regions of DEGs expressed in 968Rb. Enriched categories include defense response, regulation of transcription, and response to biotic stimulus, indicating transcriptional activation of resistance-associated genes prior to coding sequence.

Supplementary Figure S6. GO term enrichment of downstream regions in 968Rb. Bar polar plot representing enriched GO terms within downstream regions of DEGs in 968Rb. The results suggest enrichment of post-transcriptional regulation and signaling components potentially involved in modulating the plant's immune response and recovery processes.

Supplementary Figure S7. GO term enrichment of upstream regions in True French. Bar polar plot of enriched GO categories in upstream regions of DEGs from the susceptible True French cultivar. Compared to 968Rb, the enrichment is less pronounced and centered around metabolic processes and stress responses, potentially indicating a delayed or less specific defense activation.

Supplementary Figure S8. GO term enrichment of downstream regions in True French. Bar polar plot of enriched GO categories in downstream regions of DEGs from the susceptible True French cultivar. Although some categories related to signaling and metabolism are present, the overall enrichment is lower than in 968Rb, reflecting a weaker or non-specific transcriptional response following P. xanthii challenge.
