# Supplementary figures and images for: AI-Integrated Omics Analysis Reveals Cultivar-Specific Resistance Mechanisms to Powdery Mildew in Cucurbita pepo
Source: Int J Mol Sci. 2025 Nov 27;26(23):11488. doi: 10.3390/ijms262311488 (PMC12692380; doi:10.3390/ijms262311488)

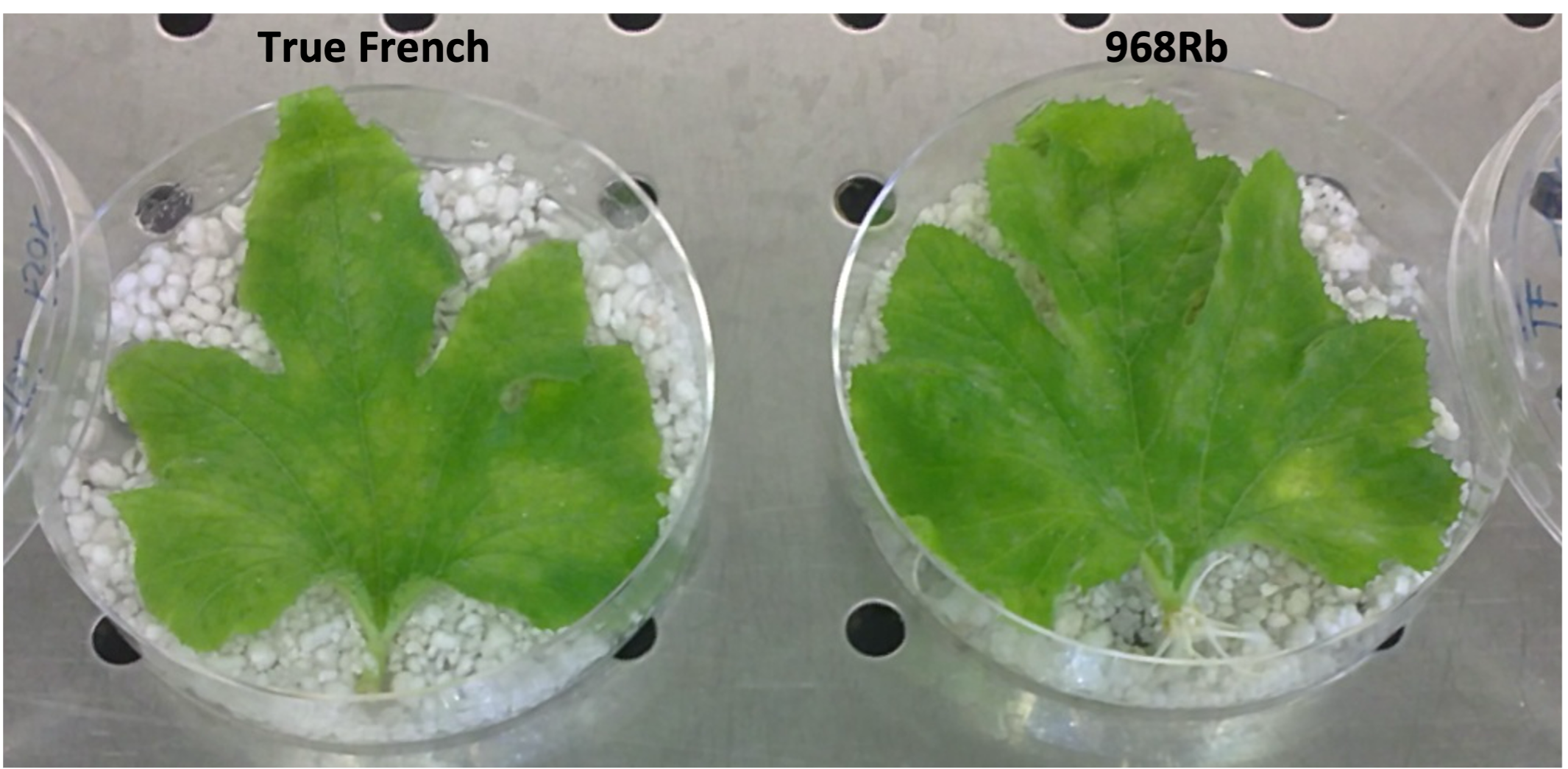

Supplement: Supplementary file 1 [file ijms-26-11488-s001.zip › Figure_S1.png]

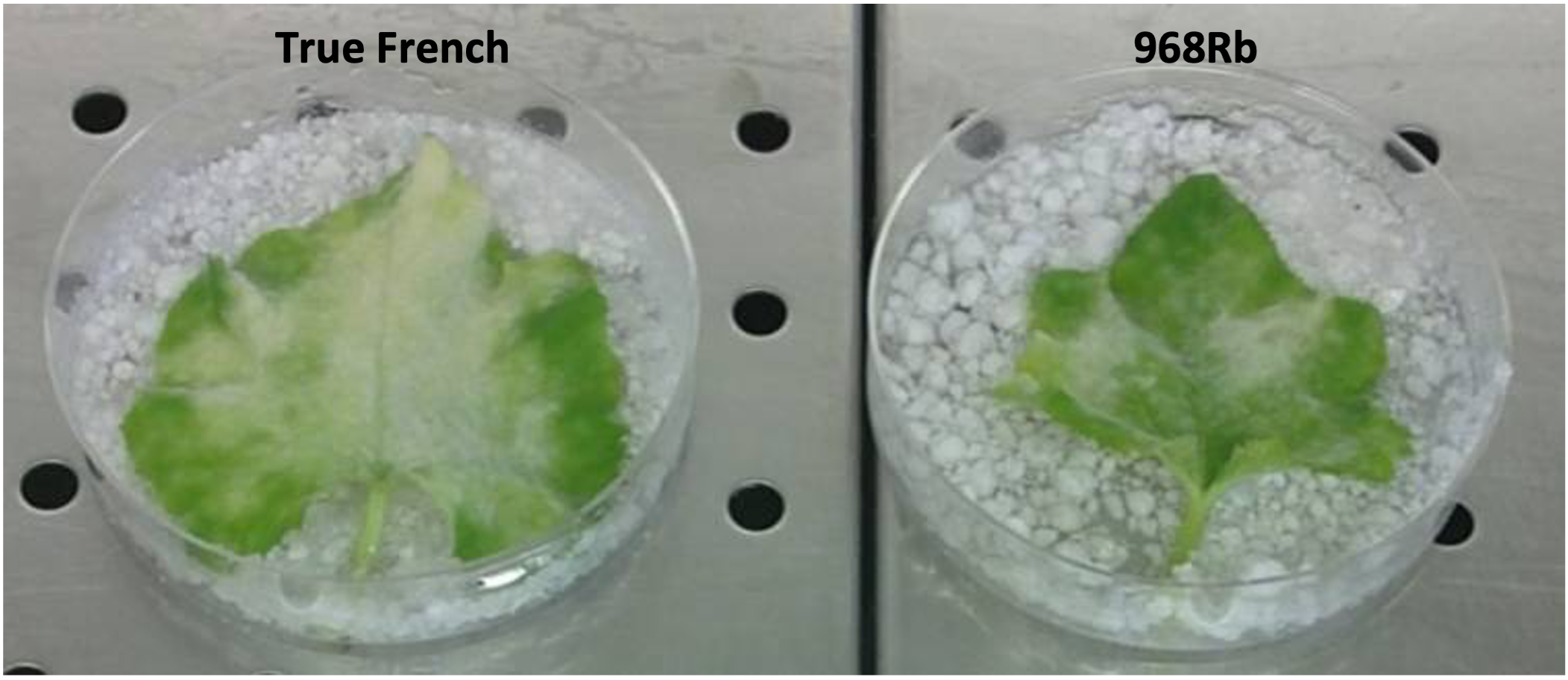

Supplement: Supplementary file 1 [file ijms-26-11488-s001.zip › Figure_S2.png]

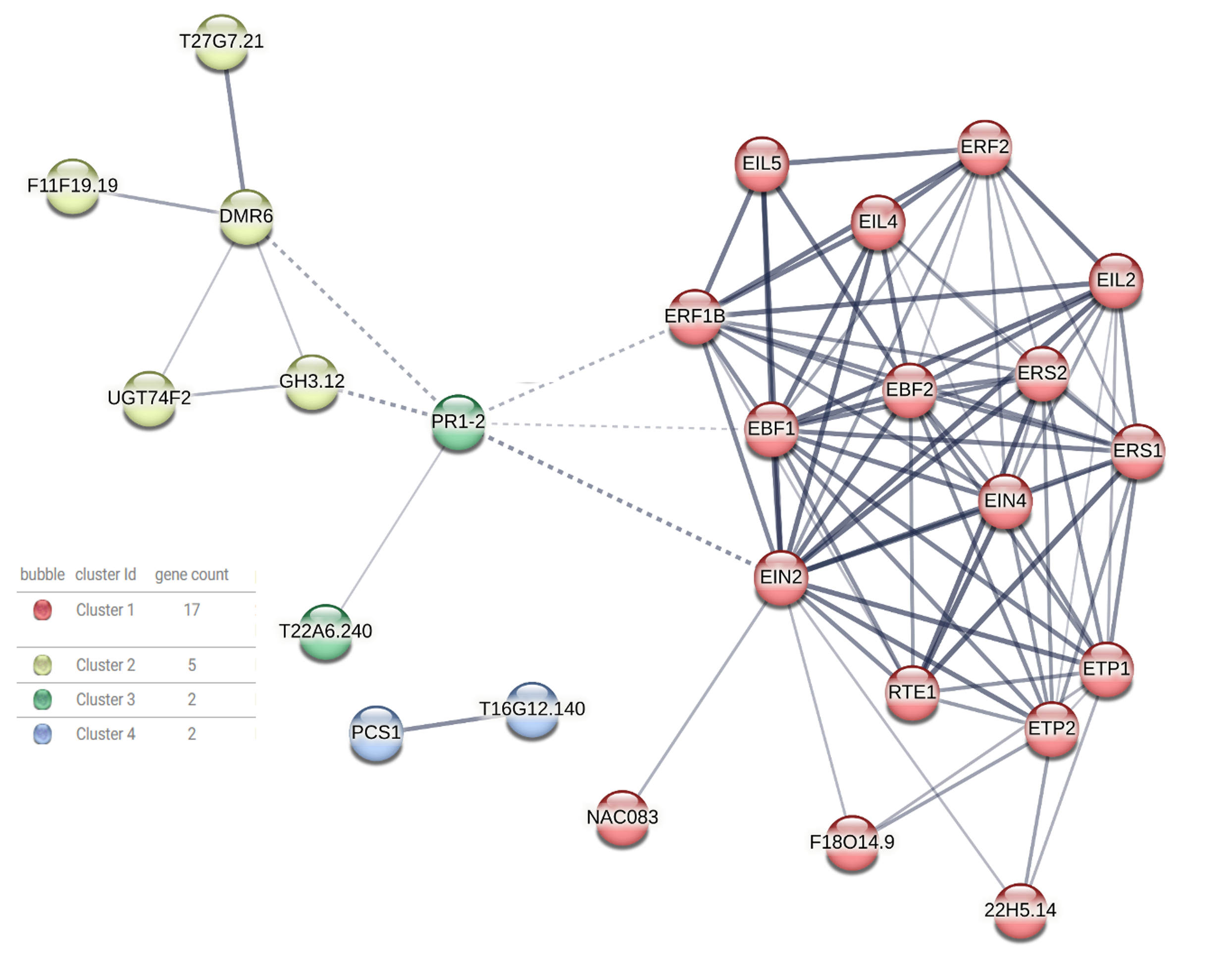

Supplement: Supplementary file 1 [file ijms-26-11488-s001.zip › Figure_S3.png]

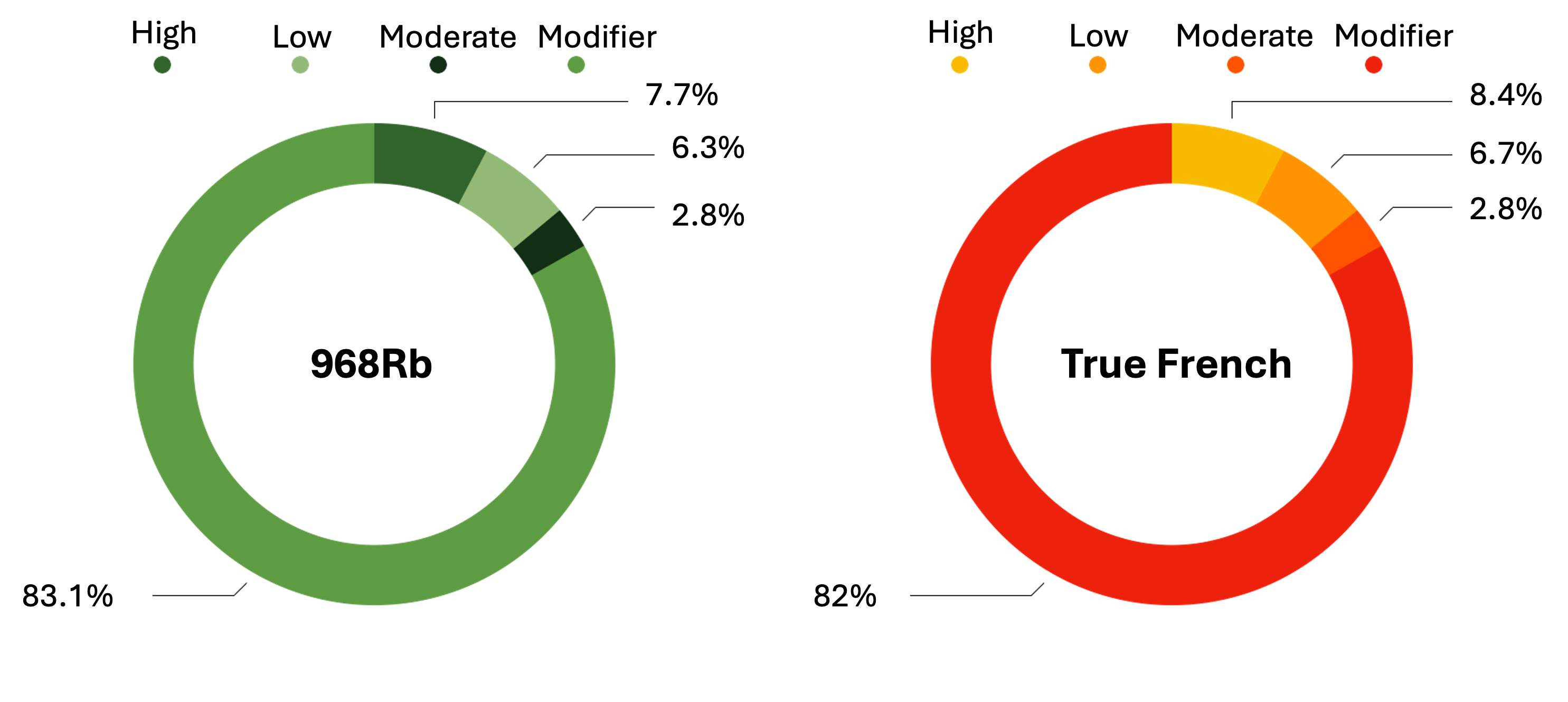

Supplement: Supplementary file 1 [file ijms-26-11488-s001.zip › Figure_S4.png]

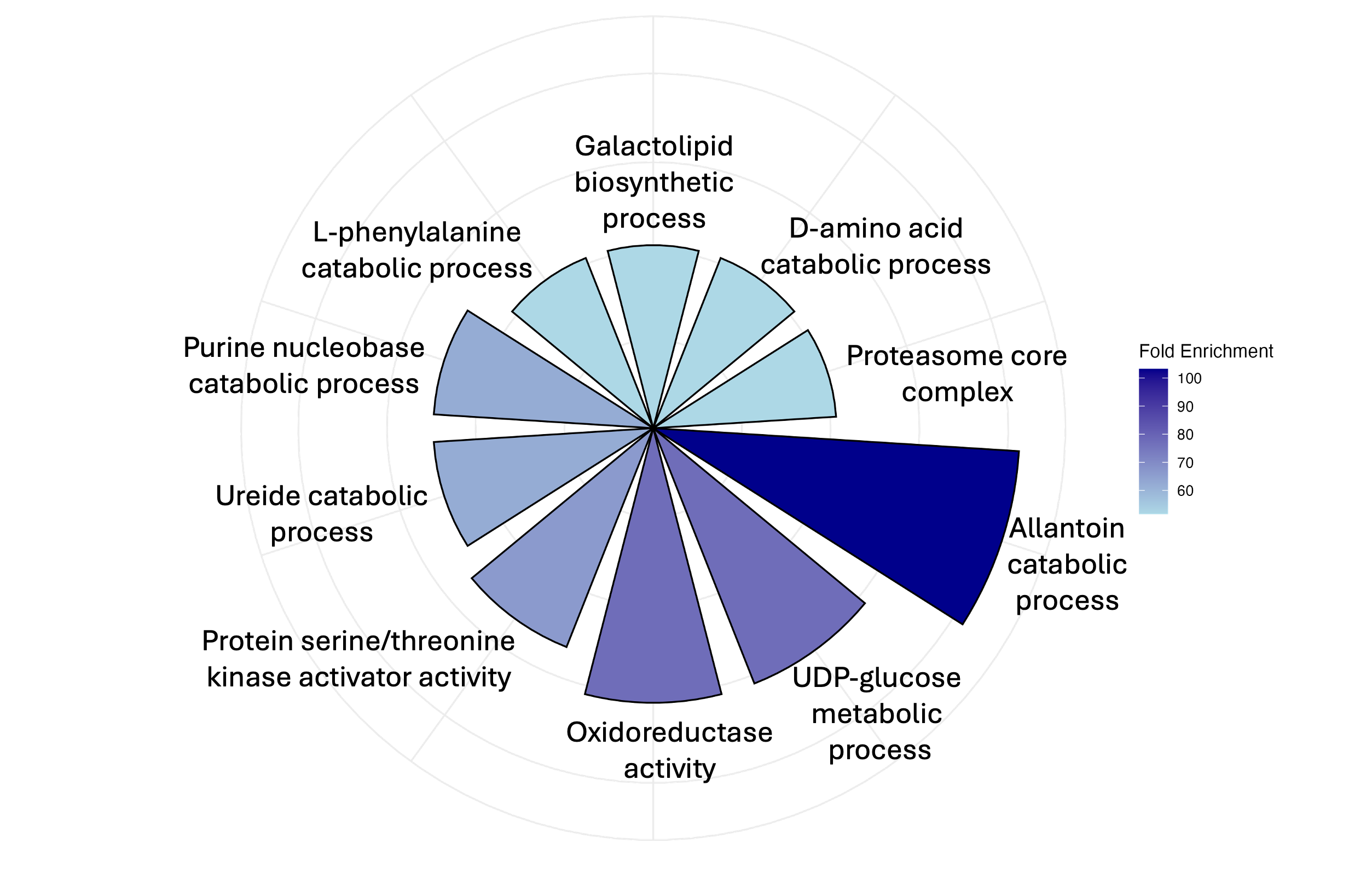

Supplement: Supplementary file 1 [file ijms-26-11488-s001.zip › Figure_S5.png]

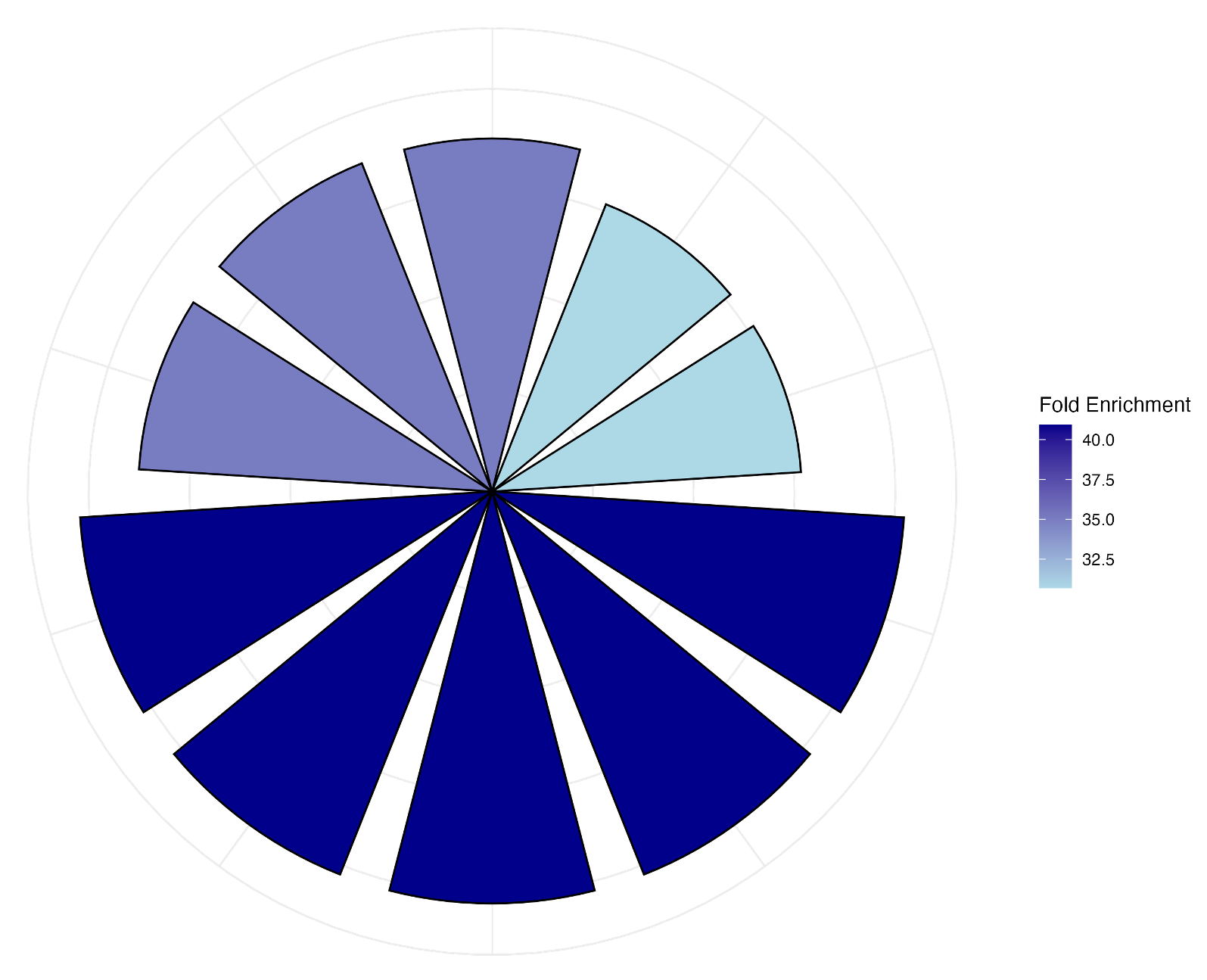

Supplement: Supplementary file 1 [file ijms-26-11488-s001.zip › Figure_S6.png]

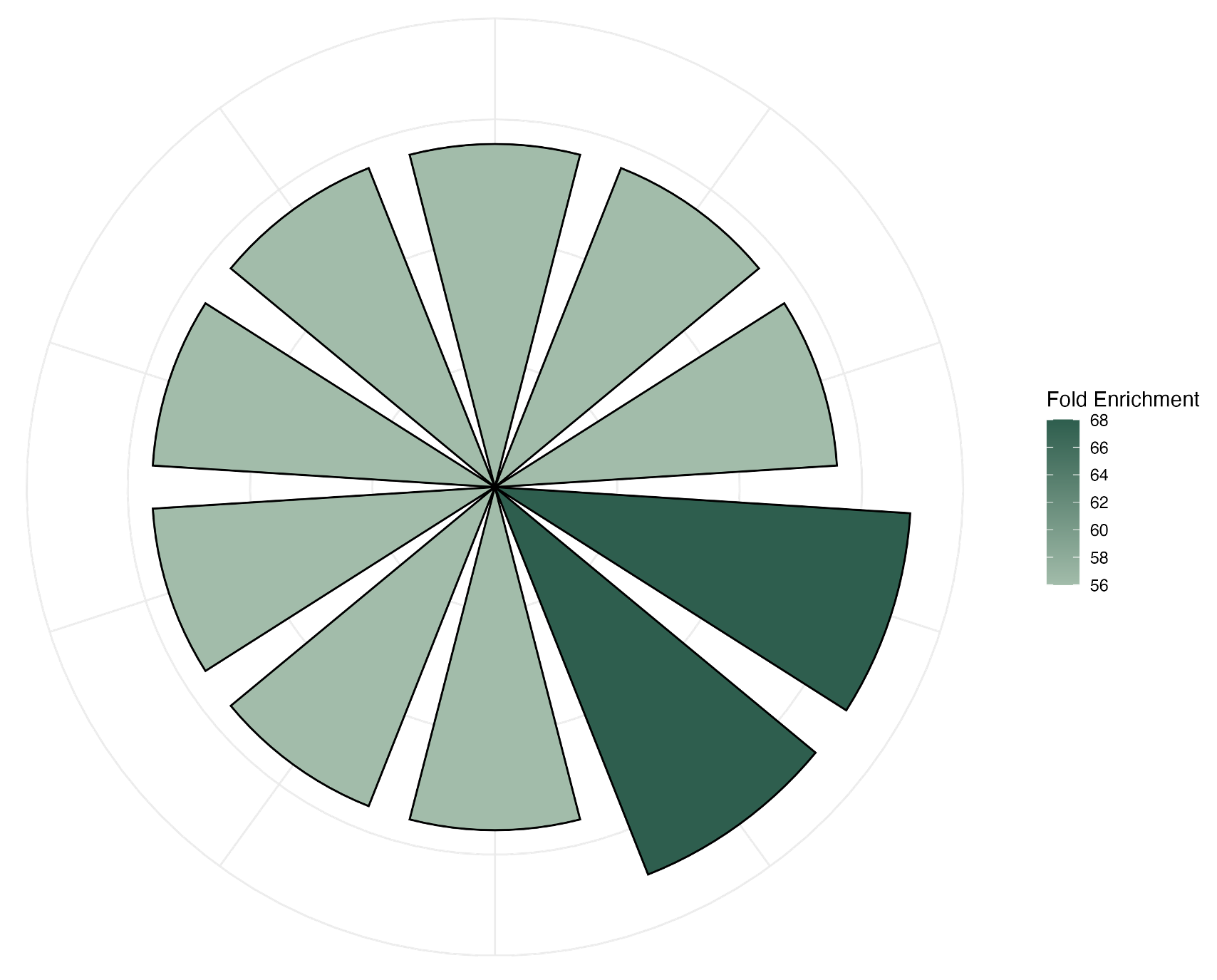

Supplement: Supplementary file 1 [file ijms-26-11488-s001.zip › Figure_S7.png]

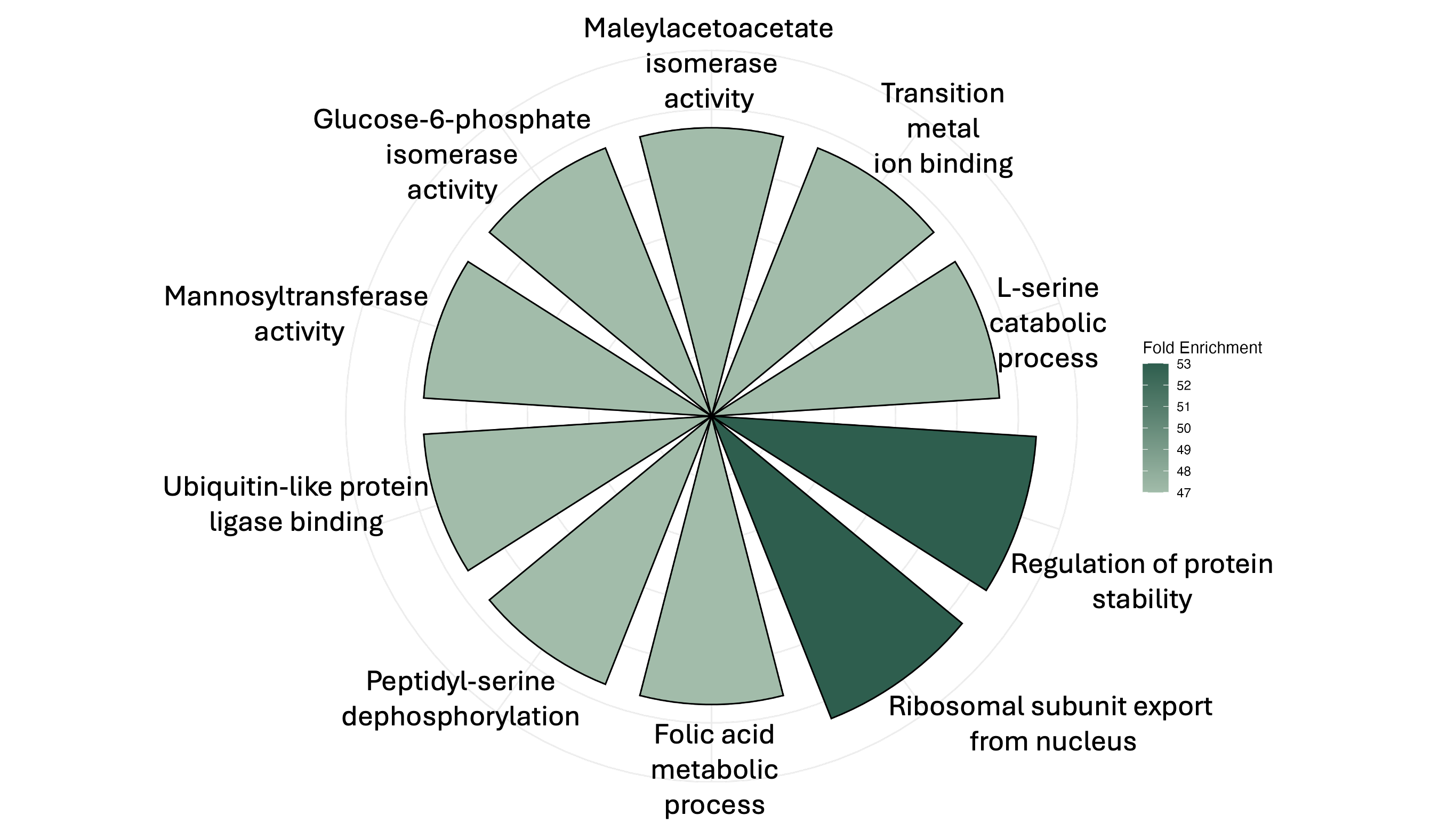

Supplement: Supplementary file 1 [file ijms-26-11488-s001.zip › Figure_S8.png]
